# Outcomes of liver transplantation for non-alcoholic steatohepatitis: A European Liver Transplant Registry study

**DOI:** 10.1016/j.jhep.2019.04.011

**Published:** 2019-08

**Authors:** Debashis Haldar, Barbara Kern, James Hodson, Matthew James Armstrong, Rene Adam, Gabriela Berlakovich, Josef Fritz, Benedikt Feurstein, Wolfgang Popp, Vincent Karam, Paolo Muiesan, John O'Grady, Neville Jamieson, Stephen J. Wigmore, Jacques Pirenne, Seyed Ali Malek-Hosseini, Ernest Hidalgo, Yaman Tokat, Andreas Paul, Johann Pratschke, Michael Bartels, Pavel Trunecka, Utz Settmacher, Massimo Pinzani, Christophe Duvoux, Philip Noel Newsome, Stefan Schneeberger, European Liver and Intestine Transplant Association (ELITA)

**Affiliations:** 1 National Institute for Health Research Birmingham Biomedical Research Centre at University Hospitals Birmingham NHS Foundation Trust and the University of Birmingham, Birmingham, UK; 2 Centre for Liver and Gastroenterology Research, Institute of Immunology and Immunotherapy, University of Birmingham, Birmingham, UK; 3 Liver Unit, University Hospitals Birmingham NHS Foundation Trust, Birmingham, UK; 4 Department of Visceral, Transplant and Thoracic Surgery, Innsbruck Medical University, Innsbruck, Austria; 5 Department of Surgery, Campus Charité Mitte and Campus Virchow Klinikum, Charité – Universitätsmedizin Berlin, Berlin, Germany; 6 Berlin Institute of Health (BIH), Berlin, Germany; 7 Institute of Translational Medicine, Queen Elizabeth Hospital Birmingham, University Hospitals Birmingham, Mindelsohn Way, Birmingham, UK; 8 Hepato-Biliary Center, AP-HP Paul Brousse Hospital, University of Paris-Sud, Inserm U776, Villejuif, France; 9 Division of Transplantation, Department of Surgery, Medical University of Vienna, Währinger Gürtel, Vienna, Austria; 10 Department of Medical Statistics, Informatics and Health Economics, Innsbruck Medical University, Innsbruck, Austria; 11 King’s Liver Transplant Unit, King’s College Hospital NHS Foundation Trust, London, UK; 12 Cambridge Transplant Centre, Cambridge University Hospitals NHS Foundation Trust, Cambridge UK; 13 MRC Centre for Inflammation Research and Royal Infirmary, University of Edinburgh, Edinburgh, UK; 14 Laboratory of Abdominal Transplantation, Universitaire Zeikenhuizen Leuven, Leuven, Belgium; 15 Shiraz Organ Transplant, IJOTM Office, Namazi Hospital, Shiraz, Iran; 16 The Leeds Teaching Hospitals NHS Trust, Leeds, UK; 17 Liver Transplantation Center, Florence Nightingale Hospital, Istanbul, Turkey; 18 Department of Visceral and Transplant Surgery, University Hospital Essen, Essen, Germany; 19 Universitatsklinikum Leipzig, Chirurgische Klinik Und Poliklinik Ii Visceral, Transplantations, Thorax und Gefabchirurgie, Leipzig, Germany; 20 Institute of Clinical and Experimental Medicine, Transplant Center, Prague, Czech Republic; 21 Universitatsklinikum Jena, Allgemeine, Viszerale und Transplantationschirurgie, Jena, Germany; 22 Sheila Sherlock Liver Centre, Royal Free London NHS Foundation Trust, London, UK; 23 Service De Chirurgie Digestive, Hopital Henri Mondor, Creteil, France

**Keywords:** ELTR database, Aetiology, Long-term follow-up, Prognosis, NAFLD, NASH

## Abstract

**Background & Aims:**

Little is known about outcomes of liver transplantation for patients with non-alcoholic steatohepatitis (NASH). We aimed to determine the frequency and outcomes of liver transplantation for patients with NASH in Europe and identify prognostic factors.

**Methods:**

We analysed data from patients transplanted for end-stage liver disease between January 2002 and December 2016 using the European Liver Transplant Registry database. We compared data between patients with NASH versus other aetiologies. The principle endpoints were patient and overall allograft survival.

**Results:**

Among 68,950 adults undergoing first liver transplantation, 4.0% were transplanted for NASH – an increase from 1.2% in 2002 to 8.4% in 2016. A greater proportion of patients transplanted for NASH (39.1%) had hepatocellular carcinoma (HCC) than non-NASH patients (28.9%, *p <*0.001). NASH was not significantly associated with survival of patients (hazard ratio [HR] 1.02, *p =* 0.713) or grafts (HR 0.99; *p =* 0.815) after accounting for available recipient and donor variables. Infection (24.0%) and cardio/cerebrovascular complications (5.3%) were the commonest causes of death in patients with NASH without HCC. Increasing recipient age (61–65 years: HR 2.07, *p <*0.001; >65: HR 1.72, *p =* 0.017), elevated model for end-stage liver disease score (>23: HR 1.48, *p* = 0.048) and low (<18.5 kg/m^2^: HR 4.29, *p* = 0.048) or high (>40 kg/m^2^: HR 1.96, *p* = 0.012) recipient body mass index independently predicted death in patients transplanted for NASH without HCC. Data must be interpreted in the context of absent recognised confounders, such as pre-morbid metabolic risk factors.

**Conclusions:**

The number and proportion of liver transplants performed for NASH in Europe has increased from 2002 through 2016. HCC was more common in patients transplanted with NASH. Survival of patients and grafts in patients with NASH is comparable to that of other disease indications.

**Lay summary:**

The prevalence of non-alcoholic fatty liver disease has increased dramatically in parallel with the worldwide increase in obesity and diabetes. Its progressive form, non-alcoholic steatohepatitis, is a growing indication for liver transplantation in Europe, with good overall outcomes reported. However, careful risk factor assessment is required to maintain favourable post-transplant outcomes in patients with non-alcoholic steatohepatitis.

## Highlights

• An increasing proportion of patients are being transplanted for non-alcoholic steatohepatitis (NASH) in Europe.
• Hepatocellular carcinoma was more common in patients transplanted with NASH.
• Survival in recipients with NASH is comparable to that of other disease indications.
• Age, BMI, and advanced liver disease predicted poorer outcomes in NASH recipients.

## Graphical abstract

**Figure:**
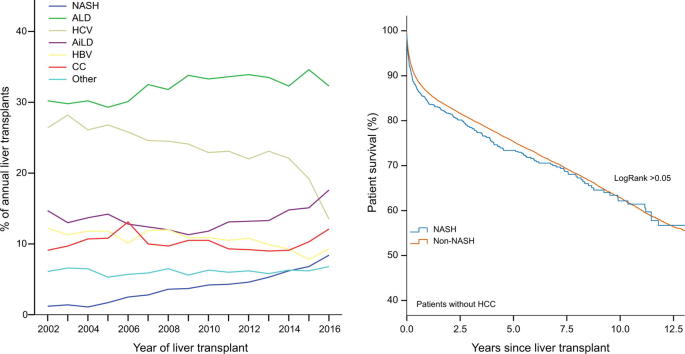

## Introduction

The prevalence of non-alcoholic fatty liver disease (NAFLD) has increased dramatically, in parallel with the worldwide increase in obesity and diabetes.[1], [2] Approximately a quarter of the European adult population have NAFLD, representing an increase of 10% since 2005.^3^

Non-alcoholic steatohepatitis (NASH) and any associated fibrosis, confer a greater risk of liver-related morbidity and mortality amongst patients with NAFLD.^4^ NASH is an increasingly common indication for liver transplantation (LT), and is now second only to alcohol-related liver disease (ALD) in the US.^5^ Similarly, NASH accounts for an increasing proportion of patients undergoing LT in the UK (4% in 1995; 12% in 2013).^6^ However, pan-European data to describe the burden of NASH on transplantation services are lacking.

Given the frequent co-existence of obesity, diabetes and related comorbidities, patients with NASH requiring LT are considered to be at a higher risk.^7^ In contrast to the US,[8], [9], [10] European reports of post-transplant outcomes of NASH have been limited to single-centre datasets.^11^ In the absence of well-validated contraindications it remains a challenge to effectively risk-stratify patients with NASH being considered for LT.^7^

We have undertaken a comprehensive analysis of LT using a prospectively updated pan-European database (n = 68,950) to determine the frequency and outcomes of patients transplanted for NASH. Building on this assessment, we have identified variables that predict a risk of poorer clinical outcome following LT for NASH.

## Patients and methods

### Study population

We performed a retrospective cohort analysis of all adult patients (>18 years old) who underwent primary LT for chronic liver disease between 1 January 2002 and 31 December 2016 using the European Liver Transplant Registry (ELTR) database. A study request was reviewed and approved by the ELTR data committee. The ELTR prospectively collects LT data from 174 centres in 33 countries and ensures data quality and validity by annual audit and cross checking with key European organ sharing organisations as previously described.[12], [13], [14]

Data were analysed for patients transplanted for ALD, hepatitis C virus infection (HCV), hepatitis B virus infection (HBV), autoimmune liver disease (AiLD) (including primary sclerosing cholangitis, primary biliary cholangitis and autoimmune hepatitis), cryptogenic cirrhosis (CC), NASH and “other” including non-B, non-C chronic viral hepatitis, polycystic liver disease, Wilson’s disease, hereditary haemochromatosis and alpha-1-antitrypsin deficiency. The cohort included patients who had hepatocellular cancer (HCC) on the background of these chronic liver diseases (ELTR database code E1: “Cancers – Hepatocellular carcinoma, cirrhosis”). The primary liver diagnosis stated in the ELTR database was used to assign diagnoses in these analyses; secondary diagnoses were disregarded unless the primary diagnosis was HCC or cryptogenic, for which the secondary diagnosis was considered as the primary. Furthermore, patients with a primary diagnosis of NASH and a secondary diagnosis of ALD in the ELTR database were assigned a diagnosis of ALD for the study.

For the purposes of this study, patients coded as having cryptogenic disease (ELTR database codes D10 “Cirrhosis – other cirrhosis specify”, D11 “Cirrhosis – cryptogenic unknown cirrhosis” and E1 [as above] without a second diagnosis in the ELTR) were designated as “presumed” NASH if their body mass index (BMI) was ≥30 kg/m^2^, or CC if their BMI was <30 kg/m^2^. As such, the NASH cohort comprised patients with “pure” NASH, defined as those coded as NASH in the ELTR database (F91: “Metabolic disease – NASH”), and those with “presumed” NASH as described above.[15], [9], [10]

Recipient factors analysed included age at transplant, sex, height, weight, BMI, blood group, primary liver diagnosis, presence of HCC, serum creatinine, serum bilirubin, international normalized ratio (INR) and the model for end-stage liver disease (MELD) score. Creatinine, bilirubin and INR had high frequencies of missing data (>50%), and thus were not used as independent variables in analyses but contributed to MELD when possible. Of note, other metabolic risk factors including smoking, type 2 diabetes, hypertension, hyperlipidaemia and a prior history of ischaemic heart disease were not included in the dataset. Donor factors included age at death/donation, sex, BMI, blood group, and type of donor (donation after circulatory death (DCD), donation after brainstem death, living related donor, domino donor).

Outcome domains comprised of patient and graft survival status, re-transplant rates, duration of follow-up and causes of death, as coded in the ELTR database. Primary causes of death were used for analyses. Secondary, tertiary and un-coded free-text causes of death were considered if the primary cause was coded as other or unknown.

Only data from a patient’s first LT were analysed.

Patient and overall allograft survival were the principle endpoints. Overall allograft survival was calculated from the date of primary LT to the date of re-transplantation or date of death (event) or the date of last follow-up during the period when the transplant was still functioning (censored). Death-censored graft survival was not reported due to the high proportion of deaths from unknown causes (28.7% overall; 42.2% NASH, 28.2% non-NASH) which may have made this outcome subject to informative censoring.

For the purpose of survival outcome analyses and cause of death analyses, patients were subdivided into cohorts defined by the presence or absence of concomitant HCC.

The primary comparison of interest was between patients transplanted for NASH and those transplanted for other indications (non-NASH).

### Statistical analysis

Statistical analyses were performed using IBM SPSS Statistics for Windows version 24 (IBM Corp., Armonk, N.Y., USA), with *p* values of <0.05 deemed significant throughout.

Parametric continuous variables were summarised with means and standard deviations, and groups compared by independent Student’s *t* test, whereas non-parametric continuous variables were summarised by median and interquartile range, and groups compared by Mann-Whitney *U* test. Categorical variables were summarised with frequencies and percentages, and groups compared by chi-squared test.

Survival outcomes were compared between groups using Kaplan-Meier curves and log-rank tests. Hazard ratios were calculated using univariable Cox regression models. Multivariable Cox regression models were produced to determine whether NASH was independently predictive of patient outcome, after accounting for other confounding factors. A backwards stepwise approach was used to select factors for inclusion in the final model, whereby variables with a significance of *p* >0.10 were iteratively excluded from the input model. All available and clinically relevant factors were included in the input models. Where NASH was excluded due to non-significance, it was added into the final model alongside the factors identified as significant by the stepwise procedure. The analysis was then repeated for the subgroup of patients transplanted for NASH, to identify independent predictors of patient survival in this cohort.

For the purposes of Cox regression analyses, continuous variables were converted to categorical fractions based on conventional thresholds (*e.g.* World Health Organization classification of BMI), or to yield relatively equal numbers of patients in each bracket (in the absence of a widely accepted convention). Hazard ratios (HR) from regression analyses were expressed relative to a reference category defined either by the group that was closest to physiological normal, the group estimated to have the lowest associated mortality or the largest group (HR = 1). Specifically, for the model that was produced to identify independent predictors of patient survival in patients with NASH, risk was assigned against a recipient BMI of 25–30 kg/m^2^ (n = 233), rather than 18.5–25 kg/m^2^ (n = 71) due to there being significantly fewer patients in that bracket, and the recognised curvilinear association between BMI and mortality, whereby the perceived lowest risk has shifted to a value between 25 and 30 kg/m^2^ in more recent years.^17^

Cases with missing data were excluded on a per-analysis basis. However, 3 key variables had a significant number of missing values – MELD (31.4%), recipient BMI (33.7%), and donor BMI (30.2%). We ensured maximal case inclusion in the multivariable analyses by including the cases with the missing values by assigning them to a separate “missing” category.

The frequencies of deaths due to specific causes were apportioned relative to the total number of deaths in patients transplanted for a specific indication. Cause-specific survival analyses were then performed using univariable Cox regression models, with comparisons between NASH and non-NASH recipients.

## Results

### Prevalence of NASH as an indication for liver transplantation over time

**Fig. 1:**
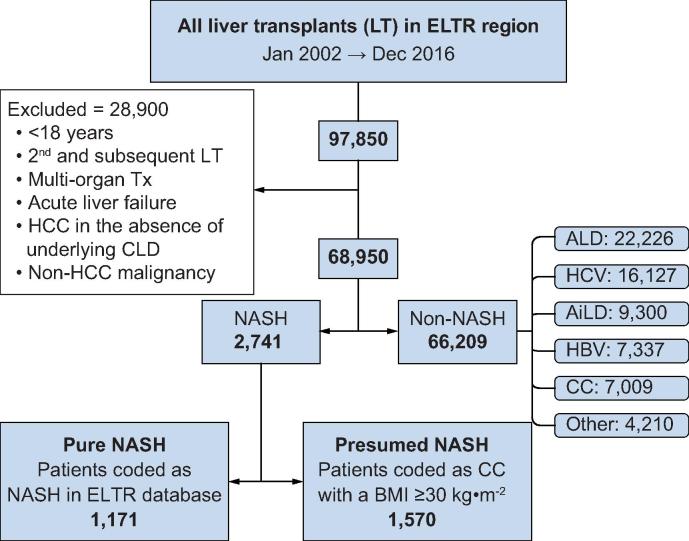
**Flow chart of case selection from the ELTR database.** AiLD, autoimmune liver disease; ALD, alcohol-related liver disease; CC, cryptogenic cirrhosis; CLD, chronic liver disease; ELTR, European Liver Transplant Registry; HBV, hepatitis B virus infection; HCC, hepatocellular carcinoma; HCV, hepatitis C virus infection; NASH, non-alcoholic steatohepatitis.

**Fig. 2:**
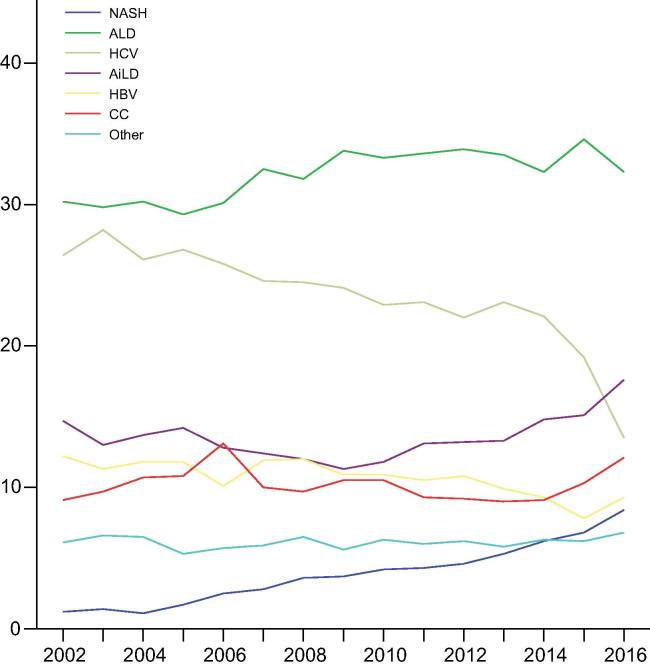
**Trends of annual primary liver transplants performed for different indications in the ELTR region.** AiLD, autoimmune liver disease; ALD, alcohol-related liver disease; CC, cryptogenic cirrhosis; ELTR, European Liver Transplant Registry; HBV, hepatitis B virus infection; HCV, hepatitis C virus infection; NASH, non-alcoholic steatohepatitis. (This figure appears in colour on the web.)

After exclusions, 68,950 patients underwent a primary LT for chronic liver disease in the study period (Fig. 1). NASH was the primary indication in 2,741 patients (4.0%), and ALD was the most common indication (22,226; 32.2%). The proportion of transplants performed for patients with NASH increased significantly over time from 1.2% in 2002 to 8.4% in 2016 (*p <*0.001) (Fig. 2, Table S1).

### Characteristics of transplant recipients and donors

In comparison to patients transplanted for other indications (Table 1), recipients with NASH were older (median: 60 *vs.* 55 years, *p <*0.001) and had a greater BMI (mean: 32.6 kg/m^2^ *vs.* 25.8 kg/m^2^, *p <*0.001). HCC was more common in recipients transplanted for NASH (39.1% *vs.* 28.9%, *p <*0.001). Moreover, the proportion of patients with underlying NASH amongst those transplanted with HCC increased from 1.3% in 2002–2004, to 8.3% in 2014–16 (*p <*0.001) (Fig. S1). Patients with NASH received organs from donors who were marginally older (median: 53 *vs.* 52 years, *p =* 0.030), more likely to be male (62.3% *vs.* 57.6%, *p <*0.001) and of a greater BMI (26.9 kg/m^2^ *vs.* 25.5 kg/m^2^, *p <*0.001) and received more DCD organs (6.6% *vs.* 2.6%, *p <*0.001). However, after adjusting for the increase in use of DCD organs over time, the rates of DCD use were similar in transplants for NASH and non-NASH indications (odds ratio [OR] 1.53; 0.21–11.11; *p =* 0.677; Table S2). Subgroup analyses divided by patients with and without HCC identified similar recipient and donor differences between NASH and non-NASH groups (Table S3).

**Table 1:** **Comparison of donor and recipient factors in patients transplanted for NASH and non-NASH indications.**

	**NASH** n = 2,741	**Non-NASH** n = 66,209
**Recipient characteristics**		
Age, years, median (IQR)**	60 (54–64)	55 (48–61)
Sex, male, %	71.1	72.1
Blood group, %		
A	43.6	43.6
AB	5.8	5.6
B	13.0	12.7
O	37.6	38.1
BMI, kg/m^2^, mean (SD)**	32.6 (4.6)	25.8 (4.4)
MELD, median (IQR)	16 (12–21)	16 (12–22)
HCC, % **	39.1	28.9
**Donor characteristics**		
Age, median (IQR)*	53 (39–61)	52 (37–64)
Sex, male, %**	62.3	57.6
Blood group, *%*		
A	41.8	42.7
AB	4.5	4.2
B	11.2	11.7
O	42.5	41.4
BMI, kg/m^2^, mean (SD)**	26.9 (4.8)	25.5 (4.3)
Type of donor, %**		
DBD	84.6	90.4
DCD	6.6	2.6
Domino	0.8	1.0
Living	8.0	6.1

Values in bold denote significance. *p <0.05; **p <0.001. BMI, body mass index; DBD, donation after brainstem death; DCD, donation after circulatory death; HCC, hepatocellular carcinoma; MELD, model for end-stage liver disease; NASH, non-alcoholic steatohepatitis.

Parametric continuous variables were summarised with means and standard deviations, and groups compared by independent Student’s *t* test, whereas non-parametric continuous variables were summarised by median and interquartile range, and groups compared by Mann-Whitney *U* test. Categorical variables were summarised with frequencies and percentages, and groups compared by chi-squared test.

### Patient survival outcomes after liver transplantation

**Fig. 3:**
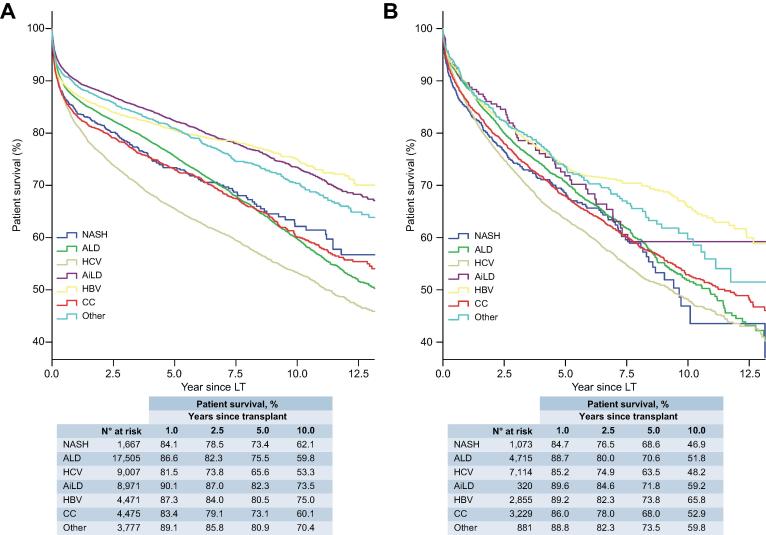
**Survival analysis for patients undergoing primary liver transplantation for different indications.** Kaplan-Meier curves of patient survival for cases without (A), and with (B) HCC (log-rank: *p <*0.001 for both). AiLD, autoimmune liver disease; ALD, alcohol-related liver disease; CC, cryptogenic cirrhosis; ELTR, European Liver Transplant Registry; HBV, hepatitis B virus infection; HCV, hepatitis C virus infection; LT, liver transplant; NASH, non-alcoholic steatohepatitis. (This figure appears in colour on the web.)

**Fig. 4:**
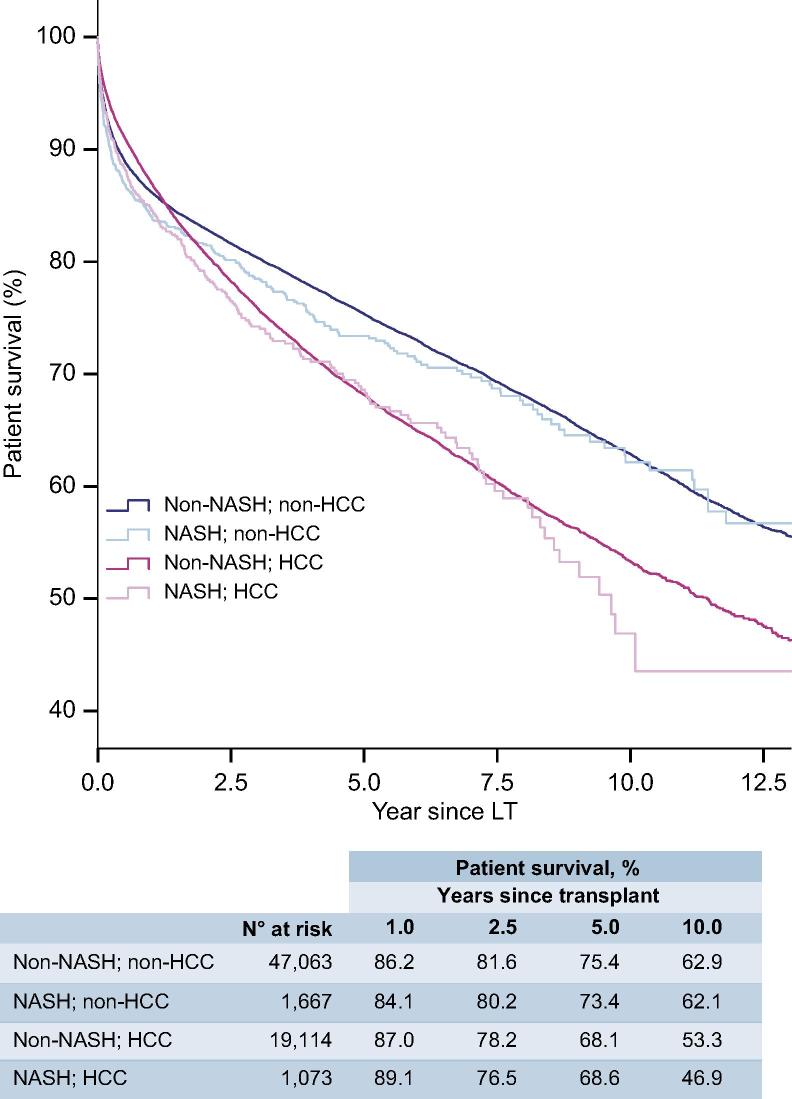
**Survival analysis for patients with and without HCC undergoing primary LT for NASH and non-NASH indications.** Kaplan-Meier analysis demonstrated no significant survival differences between patients transplanted for NASH and non-NASH indications amongst those without HCC (log-rank: *p* = 0.081) or with HCC (log-rank: *p =* 0.155). There is a significant difference between patients transplanted with and without HCC overall (log-rank: *p <*0.001). HCC, hepatocellular carcinoma; LT, liver transplant; NASH, non-alcoholic steatohepatitis. (This figure appears in colour on the web.)

There was no significant difference in post-LT patient survival between NASH and non-NASH recipients (Fig. 4), either for recipients without (HR 1.10; 95% CI 0.99–1.22; Table 2) or with HCC (HR 1.09; 95% CI 0.97–1.23; Table S4). Amongst those without HCC, recipients with NASH (n = 1,667) had equivalent post-LT survival to patients with ALD (n = 17,505; HR 0.95; 95% CI 0.85–1.06) and better survival than those with HCV (n = 9,007; HR 1.27; 95% CI 1.14–1.42; *p <*0.001) (Table 2, Fig. 3A). For those with HCC, survival in recipients with NASH (n = 1,073) was marginally worse than ALD (n = 4,715; HR 0.87; 95% CI 0.76–0.99; *p =* 0.034), but was similar to HCV (n = 7,114; HR 1.07; 95% CI 0.94–1.21) and CC (n = 3,229; HR 0.93; 95% CI 0.81–1.06) (Table S4, Fig. 3B).

**Table 2:** **Recipient and donor factors that influence patient survival in transplant recipients without HCC.**

	**Univariable**	**Multivariable**
	**HR (95% CI)**	
**Recipient characteristics**		
NASH (*vs*. non–NASH)	1.10 (0.99–1.22)	0.97 (0.86–1.09)
Cirrhosis aetiology	Overall**	n.a.
NASH	1.00	
ALD	0.95 (0.85–1.06)	
HCV	1.27 (1.14–1.42)**	
AiLD	0.62 (0.56–0.70)**	
HBV	0.68 (0.60–0.77)**	
CC	1.01 (0.90–1.14)	
Other	0.70 (0.62–0.80)**	
Age (years)	Overall**	Overall**
≤45	1.00	1.00
46–55	1.24 (1.18–1.31)**	1.24 (1.18–1.31)**
56–60	1.46 (1.38–1.55)**	1.49 (1.41–1.59)**
61–65	1.74 (1.64–1.85)**	1.78 (1.67–1.89)**
>65	1.94 (1.80–2.09)**	2.04 (1.89–2.20)**
Sex, male	1.12 (1.07–1.16)**	1.11 (1.06–1.15)**
MELD	Overall**	Overall**
≤11	1.00	1.00
>11, ≤14	0.97 (0.89–1.07)	0.98 (0.89–1.07)
>14, ≤18	0.99 (0.91–1.07)	1.02 (0.94–1.11)
>18, ≤23	1.00 (0.92–1.09)	1.05 (0.96–1.15)
>23	1.48 (1.37–1.60)	1.52 (1.40–1.64)**
Missing value	1.15 (1.07–1.24)	1.22 (1.12–1.33)**
Blood group	Overall	Overall*
A	1.00	1.00
AB	0.98 (0.90–1.06)	1.18 (1.01–1.38)*
B	0.99 (0.94–1.05)	1.12 (0.95–1.31)
O	1.00 (0.96–1.04)	0.94 (0.84–1.07)
BMI (kg/m^2^)	Overall**	Overall**
≤18.5	1.20 (1.06–1.36)*	1.34 (1.18–1.52)**
>18.5, ≤25.0	1.00	1.00
>25.0, ≤30.0	1.00 (0.95–1.06)	0.95 (0.90–1.01)
>30.0, ≤35.0	1.09 (1.02–1.17)*	1.04 (0.97–1.12)
>35.0, ≤40.0	1.09 (0.96–1.23)	1.06 (0.93–1.21)
>40.0	1.35 (1.10–1.67)*	1.35 (1.09–1.67)*
Missing value	1.02 (0.97–1.07)	1.04 (0.98–1.11)
**Donor characteristics**		
Age (years)	Overall**	Overall**
≤34	1.00	1.00
35–47	1.19 (1.13–1.27)**	1.16 (1.09–1.23)**
48–57	1.30 (1.22–1.37)**	1.25 (1.18–1.33)**
58–67	1.45 (1.37–1.54)**	1.38 (1.29–1.47)**
>68	1.63 (1.54–1.73)**	1.52 (1.43–1.62)**
Sex, male	0.99 (0.95–1.03)	not in final model
Blood group	Overall	Overall*
A	1.00	1.00
AB	0.91 (0.83–1.00)	0.82 (0.68–0.97)*
B	0.96 (0.91–1.02)	0.90 (0.76–1.06)
O	1.02 (0.98–1.06)	1.07 (0.95–1.21)
BMI (kg/m^2^)	Overall**	Overall**
≤18.5	0.92 (0.80–1.06)	0.91 (0.79–1.06)
>18.5, ≤25.0	1.00	1.00
>25.0, ≤30.0	1.10 (1.05–1.15)**	1.02 (0.97–1.07)
>30.0, ≤35.0	1.09 (1.01–1.18)*	1.02 (0.94–1.11)
>35.0, ≤40.0	1.10 (0.95–1.28)	1.02 (0.88–1.19)
>40.0	0.89 (0.69–1.15)	0.85 (0.66–1.10)
Missing value	0.92 (0.88–0.96)	0.85 (0.80–0.90)**
Type of donor	Overall**	Overall**
DBD	1.00	1.00
DCD	0.68 (0.59–0.79)**	0.73 (0.62–0.85)**
Domino	1.20 (1.00–1.23)	1.20 (0.99–1.45)
Living	1.14 (1.05–1.23)**	1.43 (1.31–1.56)**
**Other variables**		
Re-transplant	1.76 (1.67–1.86)**	1.80 (1.71–1.91)**
Era of transplant	Overall**	Overall**
2002–2004	1.00	1.00
2005–2007	1.05 (0.99–1.10)	1.03 (0.98–1.09)
2008–2010	1.13 (1.07–1.19)**	1.07 (1.00–1.13)*
2011–2013	1.00 (0.94–1.06)	0.93 (0.87–0.99)*
2014–2016	0.87 (0.80–0.93)**	0.81 (0.75–0.89)**

The final multivariable models based on 47,040 patients. Values in bold denote significance. *p <0.05; **p <0.001. AiLD, autoimmune liver disease; ALD, alcohol-related liver disease; BMI, body mass index; CC, cryptogenic cirrhosis; DBD, donation after brainstem death; DCD, donation after circulatory death; HBV, hepatitis B virus infection; HCC, hepatocellular carcinoma; HCV, hepatitis C virus infection; HR, hazard ratio; MELD, model for end-stage liver disease; NASH, non-alcoholic steatohepatitis.

Hazard ratios were calculated using uni- and multivariable Cox regression models.

On multivariable Cox regression, several recipient and donor characteristics were found to be significantly associated with post-LT survival (Table 2, Table S4). Upon adjusting for these factors, NASH was not found to be a significant independent predictor of patient survival, either in patients without (HR 0.97; 95% CI 0.86–1.09) or with (HR 1.10; 95% CI 0.97–1.24) HCC. Combining the HCC groups to analyse the cohort as a whole returned similar results (HR 1.02; 95% CI 0.93–1.11) (Fig. S2 panel A, Table S5).

### Graft survival outcomes after liver transplantation

**Fig. 5:**
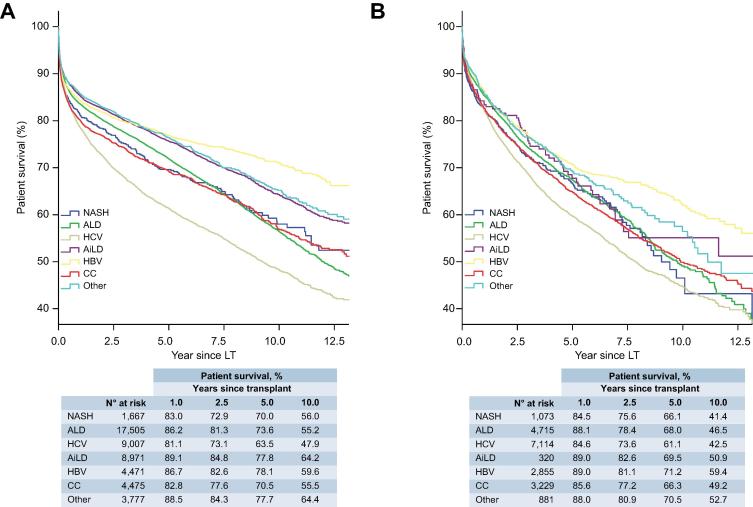
**Overall allograft survival analysis for patients undergoing primary liver transplantation for different indications.** Kaplan-Meier curves of overall allograft survival for cases without (A), and with HCC (B) (log-rank: *p <*0.001 for both). AiLD, autoimmune liver disease; ALD, alcohol-related liver disease; CC, cryptogenic cirrhosis; HBV, hepatitis B virus infection; HCV, hepatitis C virus infection; LT, liver transplant; NASH, non-alcoholic steatohepatitis. (This figure appears in colour on the web.)

On univariable analysis, post-LT graft survival (overall allograft survival) for recipients with NASH was comparable to those with non-NASH indications amongst patients without (HR 1.06; 95% CI 0.96–1.17) and with HCC (HR 1.02; 95% CI 0.91–1.15) (Fig. 5, Table S6). Analysing the cohort as a whole returned consistent results (HR 1.06; 95% CI 0.98–1.14) (Fig. S2, Table S5).

Upon adjusting for significant determinants in multivariable Cox regression analyses (Tables S5, S6), NASH was not found to be a significant independent predictor of graft survival, either in patients without (HR 0.98; 95% CI 0.88–1.09), with (HR 1.02; 95% CI 0.90–1.15), or independent of HCC (HR 0.99; 95% CI 0.91–1.08).

### Causes of death after liver transplantation

Of patients who died after LT for NASH (n = 631) and non-NASH (n = 16,989) indications, a significant proportion died from unknown causes (NASH: n = 266, 42.2%; non-NASH: n = 4,799, 28.2%; overall = 28.7%).

In recipients without HCC (Table S7), infection (n = 86, 24.0%), and cardio/cerebrovascular complications (n = 19, 5.3%) comprised the top 2 known causes of death in patients transplanted for NASH. Infection (n = 2,512, 21.6%; HR 1.15; 95% CI 0.92–1.42; *p =* 0.216) and cardio/cerebrovascular complications (n = 937, 8.1%; HR 0.70; 95% CI 0.44–1.10; *p =* 0.123) were also major causes of death in recipients transplanted for non-NASH indications, occurring at similar rates to those observed in NASH. There was a notable excess of death from extrahepatic (non-HCC) solid organ malignancy in those transplanted for ALD (n = 603, 12.9% *vs.* n = 9, 2.5%), and recurrent disease in those transplanted for HCV (n = 651, 21.5% *vs.* n = 2, 0.6%), compared to NASH recipients. These are reflected in the considerably lower risk of death from extrahepatic malignancy (HR 0.41; 95% CI 0.21–0.79; *p =* 0.008) and recurrent primary liver disease (HR 0.08; 95% CI 0.02–0.33; *p <*0.001) in NASH than in pooled non-NASH recipients.

Amongst patients with concomitant HCC (Table S8), recurrent HCC (n = 53, 19.5%), infection (n = 28, 10.3%) and extrahepatic solid organ (non-HCC) malignancy (n = 18, 6.6%) were the top 3 causes of death in patients transplanted for NASH. All 3 were also prominent causes of death in those transplanted for other indications, although there was again a notable excess risk of death from recurrence of primary (non-malignant) liver pathology in those transplanted for HCV (n = 468, 20.8%) and ALD (n = 85, 6.9%) compared to NASH recipients (n = 6, 2.2%; *p <*0.001).

### Factors that influence overall survival in patients who are transplanted for NASH

For patients transplanted for NASH in the absence of HCC, a number of recipient (age, sex, blood group, BMI, MELD) and donor (blood group) characteristics were found to be associated with post-LT survival (Table 3). Subsequent multivariable Cox regression modelling revealed that older recipient age (61–65 years: HR 2.07; 95% CI 1.39–3.08; >65 years: HR 1.72; 95% CI 1.10–2.71; relative to ≤45 years)*,* and MELD score >23 (HR 1.48; 95% CI 1.04–2.30; relative to ≤11) carried an increased risk of post-LT mortality. In addition, eccentric recipient BMI was also associated with poorer post-LT survival; an effect that was more pronounced at the extremes (≤18.5 kg/m^2^: HR 4.29; 95% CI 1.01–18.21; 18.5–25 kg/m^2^: HR 2.24; 95% CI 1.27–3.96; >40 kg/m^2^: HR 1.96; 95% CI 1.16–3.32; relative to 25–30 kg/m^2^). Male recipient gender (HR 0.79 (0.63–0.98); *p =* 0.031), and blood group B donor organs (HR 0.37; 95% CI 0.22–0.63; relative to blood group A) offered a comparative survival advantage.

**Table 3:** **Recipient and donor factors that significantly affect post-transplant survival in patients transplanted for NASH without HCC.**

	**Univariable**	**Multivariable**
	**HR (95% CI)**	
**Recipient characteristics**		
Age (years)	Overall*	Overall**
≤45	1.00	1.00
46–55	1.17 (0.79–1.76)	1.31 (0.87–1.98)
56–60	1.08 (0.71–1.62)	1.23 (0.81–1.87)
61–65	1.71 (1.16–2.52)*	2.07 (1.39–3.08)**
>65	1.50 (0.96–2.33)	1.72 (1.10–2.71)*
Sex, male	0.74 (0.60–0.92)*	0.79 (0.63–0.98)*
MELD	Overall**	Overall**
≤11	1.00	1.00
>11, ≤14	0.96 (0.63–1.48)	1.03 (0.66–1.62)
>14, ≤18	0.65 (0.43–0.98)*	0.66 (0.44–1.06)
>18, ≤23	0.68 (0.44–1.05)	0.71 (0.47–1.15)
>23	1.41 (0.97–2.05)	1.48 (1.04–2.30)*
Missing value	0.83 (0.52–1.32)	0.93 (0.57–1.51)
Blood group	Overall*	not in final model
A	1.00	
AB	0.84 (0.53–1.33)	
B	0.56 (0.37–0.85)*	
O	1.02 (0.82–1.27)	
BMI (kg/m^2^)	Overall*	Overall*
≤18.5	2.58 (0.62–10.72)	4.29 (1.01–18.21)*
>18.5, ≤25.0	1.98 (1.13–3.47)*	2.24 (1.27–3.96)*
>25.0, ≤30.0	1.00	1.00
>30.0, ≤35.0	1.22 (0.85–1.74)	1.38 (0.95–2.01)
>35.0, ≤40.0	1.38 (0.92–2.08)	1.43 (0.93–2.18)
>40.0	1.92 (1.15–3.18)*	1.96 (1.16–3.32)*
Missing value	0.89 (0.41–1.92)	1.13 (0.49–2.63)
**Donor characteristics**		
Age (years)	Overall	not in final model
≤45	1.00	
46–55	1.15 (0.82–1.62)	
56–60	1.15 (0.83–1.59)	
61–65	1.20 (0.85–1.69)	
>65	1.28 (0.88–1.84)	
Sex, male	0.97 (0.78–1.20)	not in final model
Blood group	Overall*	Overall*
A	1.00	1.00
AB	0.83 (0.49–1.41)	0.99 (0.58–1.70)
B	0.39 (0.23–0.65)**	0.37 (0.22–0.63)**
O	1.05 (0.85–1.31)	1.06 (0.85–1.32)
BMI (kg/m^2^)		not in final model
≤18.5	1.85 (0.59–5.83)	
>18.5, ≤25.0	1.13 (0.89–1.43)	
>25.0, ≤30.0	1.00	
>30.0, ≤35.0	0.98 (0.70–1.36)	
>35.0, ≤40.0	0.69 (0.34–1.40)	
>40.0	1.15 (0.54–2.45)	
Missing value	0.73 (0.44–1.20)	
Type of donor	Overall	not in final model
DBD	1.00	
DCD	0.79 (0.47–1.33)	
Domino	1.16 (0.29–4.64)	
Living	1.45 (1.02–2.05)	
**Other variables**		
Era of transplant	Overall	n.a.
2002–2004	1.00	
2005–2007	0.90 (0.59–1.37)	
2008–2010	1.18 (0.79–1.76)	
2011–2013	1.09 (0.72–1.63)	
2014–2016	1.13 (0.74–1.71)	

The final multivariable models based on 1,628 patients. Values in bold denote significance. *p <0.05; **p <0.001. BMI, body mass index; DBD, donation after brainstem death; DCD, donation after circulatory death; HCC, hepatocellular carcinoma; HR, hazard ratio; MELD, model for end-stage liver disease; NASH, non-alcoholic steatohepatitis.

Hazard ratios were calculated using uni- and multivariable Cox regression models.

For patients with NASH and concomitant HCC, none of the available variables were found to be significantly associated with survival on either univariable or multivariable analyses (Table S9).

## Discussion

This study finds the proportion of transplants for patients with NASH has risen to now account for 8.4% of annual transplants in Europe, and reflects rates published from national datasets.[18], [19] The trends are in keeping with those seen in the US where NASH accounts for more than 18% of transplants.[8], [10], [20], [21] The magnitude of the impact of NASH on transplant services in the US may forecast the future burden in Europe in heed of the projected rise of obesity across the continent.[1], [2], [22] However, wide intra-continental variations in risk factor profiles may limit the local applicability of pan-European data.[23], [24] Further to the effect of risk factors, a greater awareness of NASH, and greater confidence amongst transplant physicians to make a diagnosis based on phenotypic associations may also contribute to the greater proportion of transplants.^25^ However, the effects of ascertainment bias in our study are unlikely to be significant in the absence of a commensurate decrease in transplants performed for CC.

There remains controversy in the way large databases establish diagnoses of NASH and CC.^25^ In keeping with other large database studies, and to facilitate meaningful comparisons between datasets, we have chosen CC patients with a BMI >30 kg/m^2^ as our presumed NASH cohort.[15], [9], [10] We acknowledge that ascites and oedema contribute to the BMI and are not corrected for in the ELTR, though this is also a limitation of using other large registry databases.^8^ Of our NASH study cohort, 57.3% were “presumed NASH” which is comparable to data from the US; between 45.2%^8^ and 67.5%^9^ of the NASH cohorts using the United Network of Organ Sharing (UNOS) database were “presumed NASH”. However, the differences in the characteristics and outcomes between pure and presumed NASH (Table S10, Figs. S3, S4 and S5) highlight that NASH patients are still a heterogeneous population and systematically identifying high and low risk subsets based on recipient and donor characteristics as highlighted in Table 3, is of critical importance.

A greater proportion of recipients with NASH were transplanted for HCC than non-NASH recipients (Table 1). Our findings were in keeping with a recent analysis of the US Scientific Registry of Transplant Recipients database,^26^ in which the authors describe a 7.7-fold increase in the prevalence of NASH in patients transplanted for HCC between 2002 and 2016. A number of studies have suggested that patients with NASH are at greater risk of developing HCC,[16], [21], [27] owing partly to the risks associated with obesity and insulin resistance.[28], [29], [30] NASH-related HCC is major worldwide concern and left unchecked may offset the anticipated declines in primary liver cancer through the control of HBV and HCV.^31^

Amongst the NASH cases, there was a difference in the rate of HCC between the pure NASH (28.5%) and presumed NASH (47.1%) cohorts (Table S10), due to incorporating the orphan E1 ELTR code (Cancers – Hepatocellular carcinoma, cirrhosis; no secondary diagnosis) in to the cryptogenic cohort.^8^ The rate of HCC in CC (at the exclusion of obese patients) was 46.1% (Table S11).

As with other registry studies, readers of our data should be mindful of the potential influence of missing data points, despite the vast number of cases included in the study. As described in our Methods, a significant proportion of cases in our dataset had missing data for one of MELD, recipient BMI or donor BMI. There were statistically significant differences in patient characteristics between cases with and without missing data points (Table S12). We utilised a “missing-indicator” method to maximally utilise the available cases and minimise any loss of statistical power in our multivariable analyses. We compared patient survival in those with available data against those with missing data. The cases with a missing recipient BMI did not confer a bias to overall patient survival (univariable Cox regression; HR 1.01; 95% CI 0.98–1.04; *p =* 0.485) compared to cases with available values. Cases with missing MELD (HR 0.93; 95% CI 0.90–0.96; *p <*0.001) and donor BMI (HR 0.90; 95% CI 0.87–0.93; *p <*0.001) did carry a weighted risk, although the effect was small. Moreover, we compared multivariable models using the missing-indicator method and an “available case analysis” method whereby only cases without missing observations are included, and we found no significant differences to key outcomes (Tables S13, S14). The influence of the incomplete data is a recurrent limitation of registry database studies and although different statistical methods to account for the effect of missing data are widely used, they each carry an inherent bias. Best practice on the statistical methods should be incorporated across registries and ideally stated in the standard operating procedures of registries.

There were no significant differences in post-LT deaths due to infection and cardio/cerebrovascular events between NASH and non-NASH recipients without HCC. Conclusions drawn from these data should be tempered in consideration of the 28.7% of cases in which the causes of death were unknown. However, the loss of data quality with duration of follow-up is pervasive to large databases; an analysis of the UNOS database noted that 24% of deaths that occurred 5 years or later after transplantation were from unknown causes.^32^ The size of the database cohorts may allow for a higher tolerability towards missing data points, but it remains a limitation. By comparison, a recent meta-analysis of 6 single- and two-centre studies demonstrated an excess of deaths from sepsis (OR 1.71) and cardiovascular causes (OR 1.65) in the NASH cohort.^20^

NASH was not found to be an independent predictor of patient or graft survival. These results add to a growing body of evidence that suggest current practice in patient selection and peri-operative care results in acceptable outcomes for such patients, and reflect good utility of donor organs.[6], [10], [20] Nevertheless, our findings demand scrutiny of assessing transplant risk based on recipient age and BMI. The risk attributed to older recipients is particularly pertinent as a growing proportion of transplants are being performed on elderly recipients in both the US and Europe.^33^ Moreover, the risk carried by recipients with lower BMI may reflect the independent effects of a catabolic and sarcopenic phenotype.^34^ However, the associations at both extremes of BMI are based on relatively few patients (n = 77 for BMI ≤25 kg/m^2^; n = 92 for BMI >40 kg/m^2^, cp. n = 233 for BMI 25–30 kg/m^2^). Critical variables including pre-LT comorbidities, and in particular components of metabolic syndrome, may have influenced prognostic determinants in our analysis and significantly added to the power of our model.[11], [35] None of our measured variables were found to be associated with post-LT mortality in recipients with HCC, which suggests that HCC-specific factors that reflect the burden of disease are likely to have a critical influence on post-transplant outcomes.^36^ Tumour specific factors have only been collected by the ELTR since 2007, and a dedicated study exploring the influence of these factors on post-transplant outcomes has recently been published.^37^

Large databases such as ELTR and UNOS were designed to facilitate research but have to compromise between the practicalities of ensuring data collection and the desire to capture relevant data fields. Technological developments to optimise data collection and periodic review of collected fields in response to evolving knowledge comprise potential solutions. Moreover, harmonisation of data fields across different registries would allow meaningful comparisons between datasets.

In summary, we report a year-on-year increase in the number of LTs performed for NASH in the ELTR region since 2002. The proportion of transplants done for NASH with concomitant HCC is rising, reflecting the widely acknowledged association of NASH with HCC. NASH was not an independent predictor of post-LT patient and graft survival. Nevertheless, careful assessment and selection of patients will be critical to maintain acceptable survival in those transplanted for NASH, with specific scrutiny of female patients, recipients over the age of 60, those with advanced liver disease (MELD >23) and particularly patients with extreme high or low BMI.

## Financial support

Debashis Haldar, Matthew Armstrong, and Philip Newsome are funded/supported by the NIHR Birmingham Biomedical Research Centre [BRC-1215-20009]. This paper presents independent research supported by the NIHR Birmingham Biomedical Research Centre at the University Hospitals Birmingham NHS Foundation Trust and the University of Birmingham. Debashis Haldar is also supported by a Wellcome Trust [108741/Z/15/Z]. The views expressed are those of the author(s) and not necessarily those of the NHS, the NIHR, the Wellcome Trust or the Department of Health and Social Care.

## Conflicts of interest

The authors declare no conflicts of interest that pertain to this work.

Please refer to the accompanying ICMJE disclosure forms for further details.

## Author contributions

D Haldar: Study concept and design, analysis and interpretation of data, statistical analysis, and writing of manuscript; B Kern: Study concept and design, acquisition of data, drafting of manuscript, revision of manuscript; J Hodson: Statistical analysis and critical revision; M J Armstrong: Critical revision of the manuscript for intellectual content. Significant contribution to final revisions; R Adam: Critical revision of the manuscript for intellectual content; G Berlakovich: Critical revision of the manuscript for intellectual content, liaison person for ELTR/ELITA. J Fritz: Critical revision of the manuscript for intellectual content; B Feurstein: Critical revision of the manuscript for intellectual content; W Popp: Critical revision of the manuscript for intellectual content; V Karam: Acquisition of data, critical revision of the manuscript for intellectual content; P Muiesan: Critical revision of the manuscript for intellectual content; J O’Grady: Critical revision of the manuscript for intellectual content; N Jamieson: Critical revision of the manuscript for intellectual content; S J Wigmore: Critical revision of the manuscript for intellectual content; J Pirenne: Critical revision of the manuscript for intellectual content; S A Malek-Hosseini: Critical revision of the manuscript for intellectual content; E Hidalgo: Critical revision of the manuscript for intellectual content; Y Tokat: Critical revision of the manuscript for intellectual content; A Paul: Critical revision of the manuscript for intellectual content; J Pratschke: Critical revision of the manuscript for intellectual content; M Bartels: Critical revision of the manuscript for intellectual content; P Trunecka: Critical revision of the manuscript for intellectual content; U Settmacher: Critical revision of the manuscript for intellectual content; M Pinzani: Critical revision of the manuscript for intellectual content; C Duvoux: Critical revision of the manuscript for intellectual content; P N Newsome: Study concept and design, critical revision of the manuscript for intellectual content, study supervision; S Schneeberger: Study concept and design, critical revision of the manuscript for intellectual content, study supervision.
