# Supplementary material for: Outcomes of liver transplantation for non-alcoholic steatohepatitis: A European Liver Transplant Registry study
Source: J Hepatol. 2019 Aug;71(2):313–22. doi: 10.1016/j.jhep.2019.04.011 (PMC6656693; doi:10.1016/j.jhep.2019.04.011)
Supplement: Supplementary Data 1 [file mmc1.pdf]

Outcomes of liver transplantation for non-alcoholic steatohepatitis: A European Liver Transplant Registry study

Debashis Haldar, Barbara Kern, James Hodson, Matthew James Armstrong, Rene Adam, Gabriela Berlakovich, Josef Fritz, Benedikt Feurstein, Wolfgang Popp, Vincent Karam, Paolo Muiesan, John O'Grady, Neville Jamieson, Stephen J Wigmore, Jacques Pirenne, Seyed Ali Malek-Hosseini, Ernest Hidalgo, Yaman Tokat, Andreas Paul, Johann Pratschke, Michael Bartels, Pavel Trunecka, Utz Settmacher, Massimo Pinzani, Christophe Duvoux, Philip Noel Newsome, Stefan Schneeberger and all contributing centres (www.eltr.org) and the European Liver and Intestine Transplant Association (ELITA).

Table of contents

Fig. S1..3

Fig. S2..4

Fig. S3..5

Fig. S4..6

Fig. S5..7

Table S1..8

Table S2..9

Table S3..10

Table S4..11

Table S5..13

Table S6..15

Table S7..17

Table S8..19

Table S9..21

Table S10..23

Table S11..24

Table S12..25

Table S13..26

Table S14..28

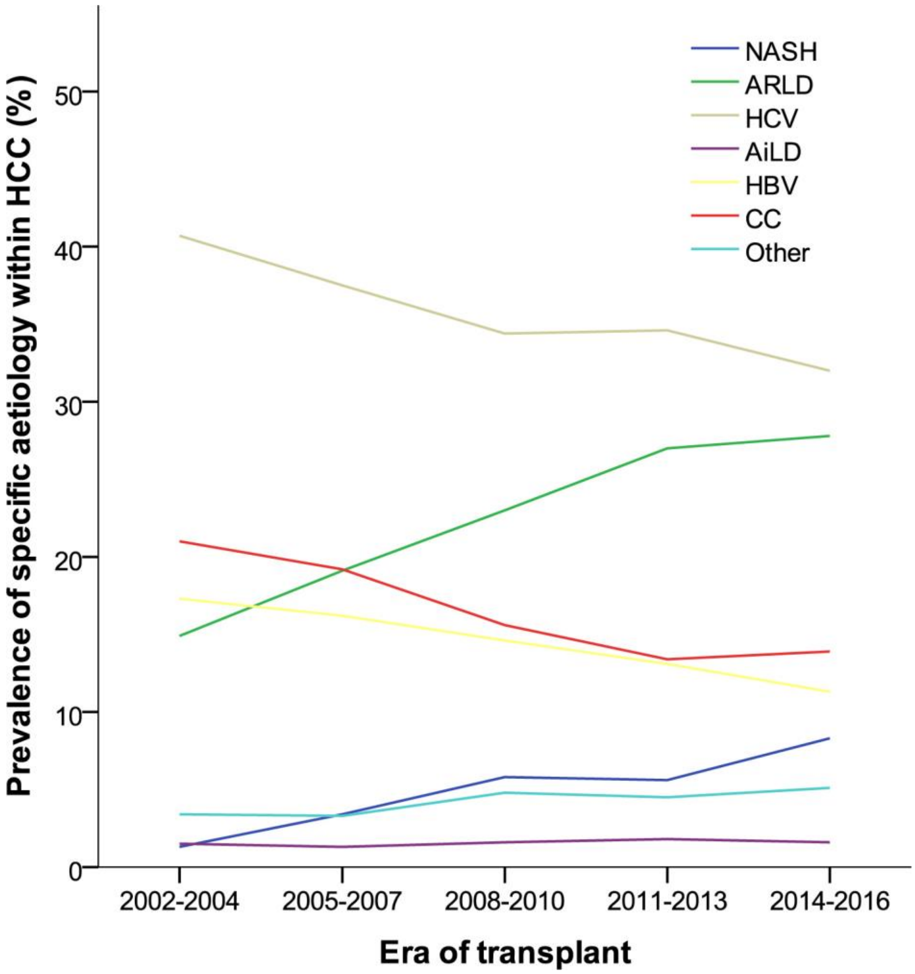

Fig. S1. Prevalence of liver disease aetiologies in patients with HCC in different era.

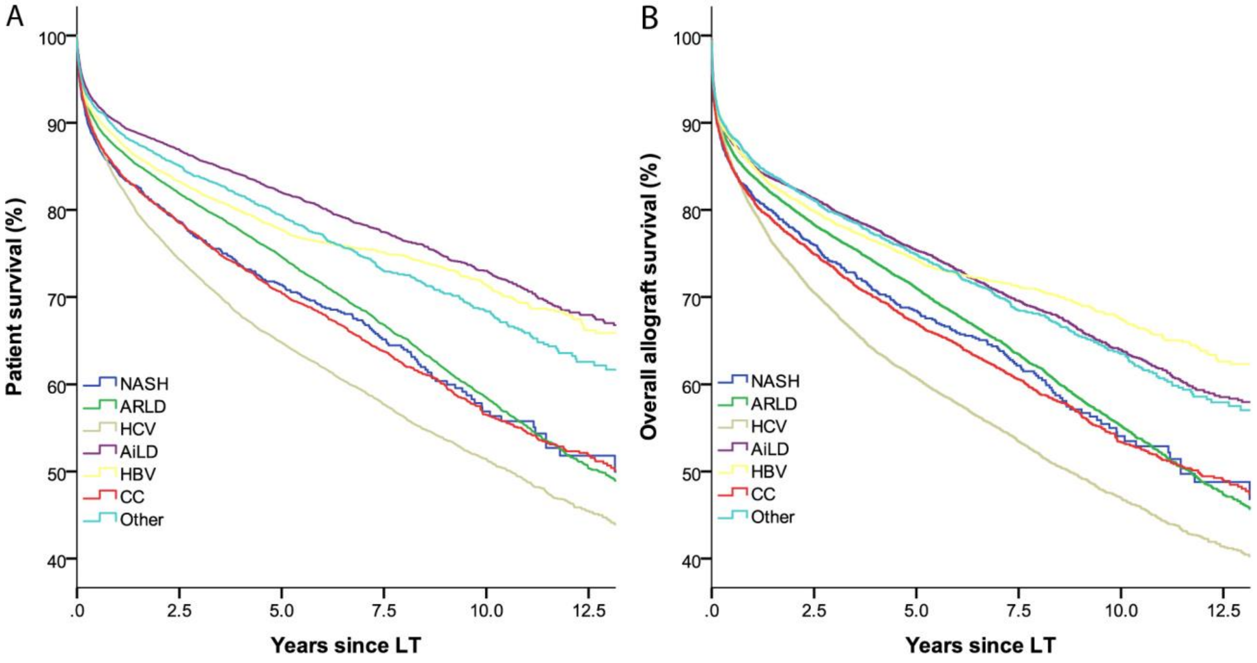

		Patient survival; %			
		Years since transplant			
	At risk; N	1.0	2.5	5.0	10.0
**NASH**	2740	84.3	78.6	71.4	56.9
**ARLD**	22220	87.0	81.9	74.6	58.5
**HCV**	16121	83.1	74.3	64.8	51.4
**AiLD**	9291	90.1	86.9	82.0	73.0
**HBV**	7330	88.0	83.2	77.7	71.3
**CC**	7006	84.6	78.5	70.5	56.5
**Other**	4209	89.0	85.0	79.4	68.3

		Patient survival; %			
		Years since transplant			
	At risk; N	1.0	2.5	5.0	10.0
**NASH**	2740	83.6	76.9	68.5	51.3
**ARLD**	22220	86.6	80.7	72.5	53.8
**HCV**	16121	82.7	73.3	62.5	45.9
**AiLD**	9291	89.1	84.7	77.5	63.7
**HBV**	7330	87.6	81.9	75.3	65.4
**CC**	7006	84.1	77.4	68.4	52.3
**Other**	4209	88.4	83.6	76.2	62.2

Fig. S2. Kaplan-Meier curves of patient survival (A) and overall allograft survival (B) analysis for all patients undergoing primary LT for different indications (log-rank: *P*<0.001 for both).

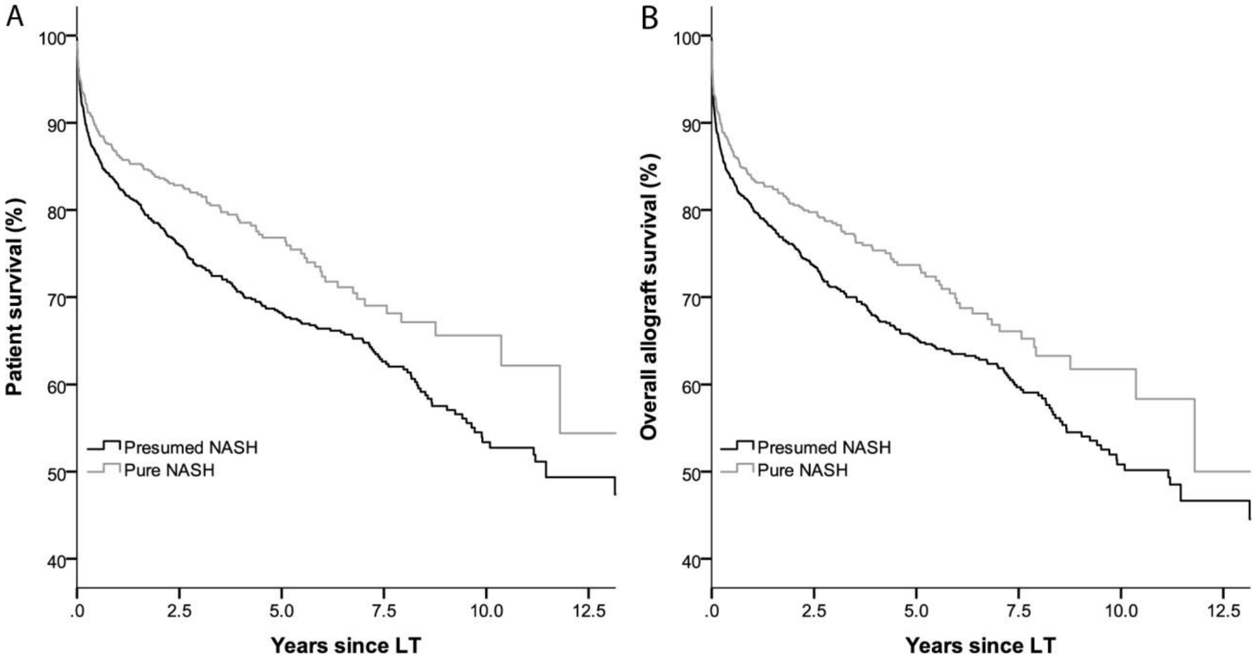

		Patient survival; %			
		Years since transplant			
	At risk; N	1.0	2.5	5.0	10.0
Presumed NASH	1570	82.9	75.9	68.2	53.4
Pure NASH	1170	86.3	82.8	76.8	65.6

		Patient survival; %			
		Years since transplant			
	At risk; N	1.0	2.5	5.0	10.0
Presumed NASH	1570	80.2	73.5	65.3	50.8
Pure NASH	1170	83.5	79.7	73.7	61.8

Fig. S3. Kaplan-Meier curves of patient survival (A) and overall allograft survival (B) analysis for patients with pure NASH and presumed NASH undergoing primary LT (log-rank: *P*<0.001 for both).

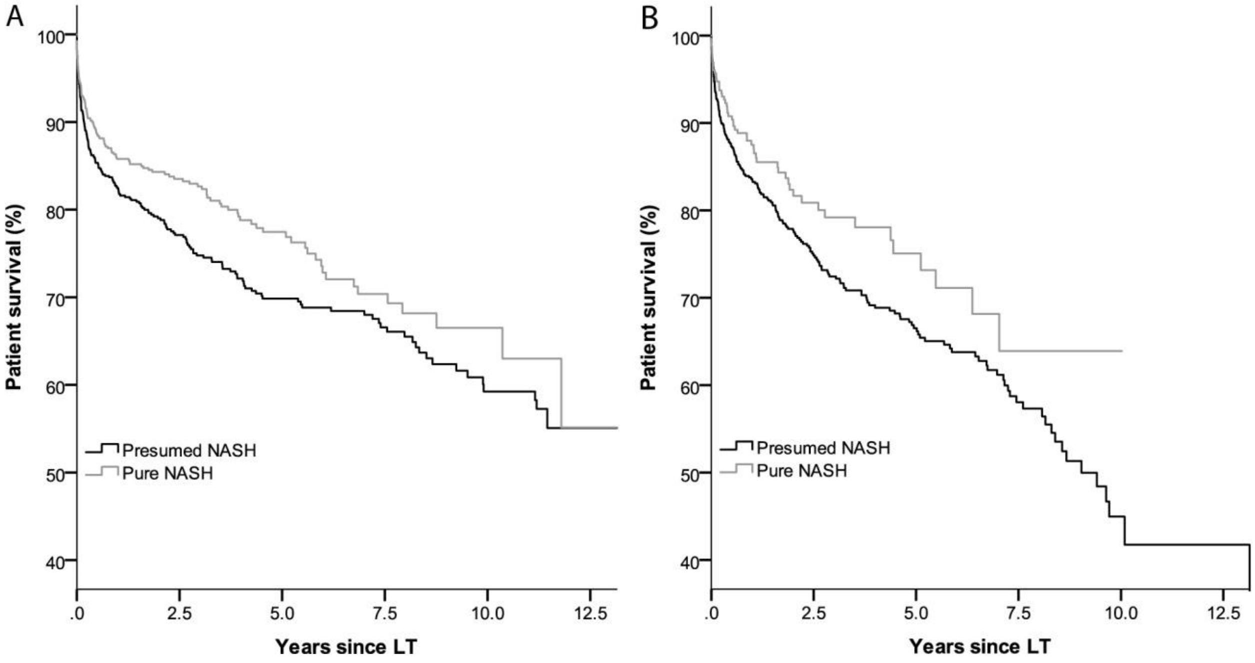

Fig. S4. Kaplan-Meier curves of patient survival comparing those transplanted for pure and presumed NASH stratified by the absence (A) and presence (B) of HCC (A: log-rank: *P*=0.019; B: log-rank *P*=0.046).

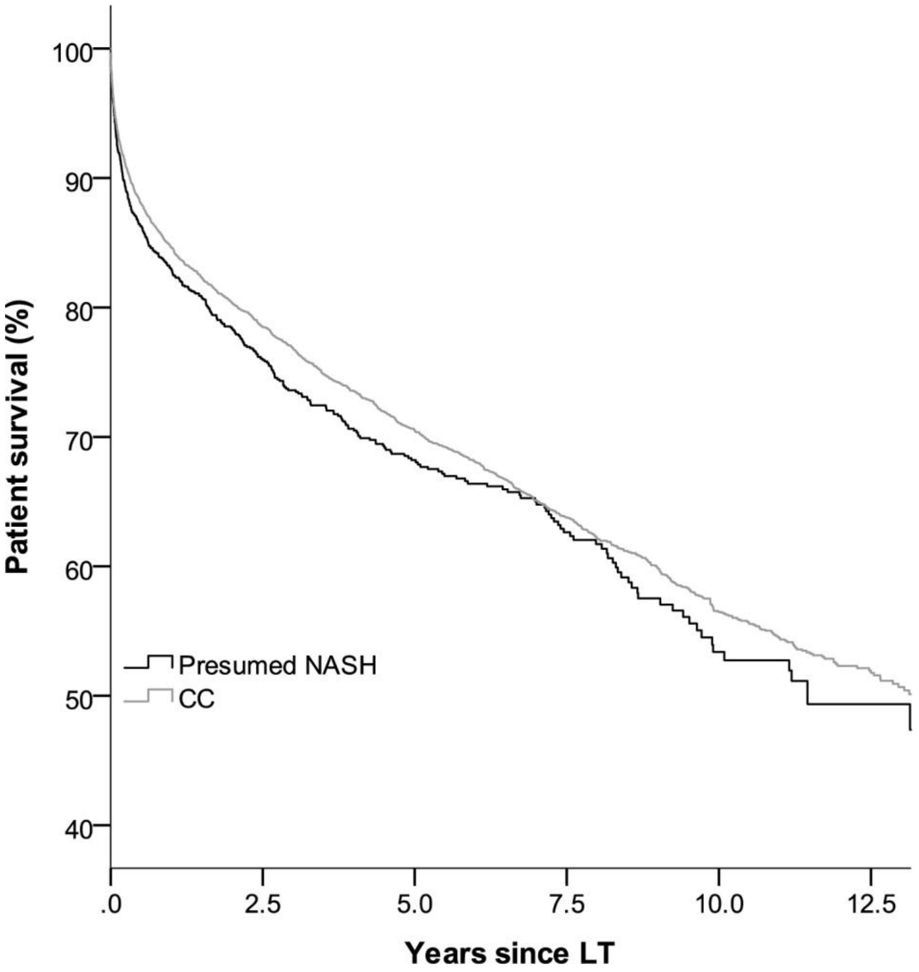

Fig. S5. Kaplan-Meier curves of patient survival comparing those transplanted for presumed NASH and those with CC (log-rank: *P*=0.072).

		Year of Transplant														
		**2002**	**2003**	**2004**	**2005**	**2006**	**2007**	**2008**	**2009**	**2010**	**2011**	**2012**	**2013**	**2014**	**2015**	**2016***	***Total***
**Indication for Transplant**	**NASH**	45 (1.2)	53 (1.4)	44 (1.1)	72 (1.7)	106 (2.4)	132 (2.8)	175 (3.6)	178 (3.7)	205 (4.2)	219 (4.3)	232 (4.6)	274 (5.3)	331 (6.2)	343 (6.8)	332 (8.4)	*2741*
	**ARLD**	1135 (30.2)	1103 (29.8)	1246 (30.2)	1257 (29.4)	1297 (30.1)	1507 (32.5)	1540 (31.8)	1620 (33.8)	1611 (33.4)	1721 (33.7)	1713 (33.9)	1727 (33.5)	1717 (32.3)	1756 (34.6)	1276 (32.3)	*22226*
	**HCV**	994 (26.4)	1047 (28.2)	1077 (26.1)	1148 (26.8)	1110 (25.8)	1139 (24.5)	1184 (24.4)	1155 (24.1)	1106 (22.9)	1180 (23.1)	1111 (22.0)	1190 (23.1)	1176 (22.1)	977 (19.2)	533 (13.5)	*16127*
	**AiLD**	553 (14.7)	482 (13.0)	567 (13.7)	610 (14.2)	550 (12.8)	573 (12.4)	582 (12.0)	543 (11.4)	568 (11.8)	672 (13.1)	667 (13.2)	686 (13.3)	785 (14.7)	768 (15.1)	694 (17.6)	*9300*
	**HBV**	458 (12.2)	418 (11.3)	488 (11.8)	506 (11.8)	436 (10.1)	552 (11.9)	579 (12.0)	523 (10.9)	529 (10.9)	539 (10.5)	547 (10.8)	508 (9.9)	493 (9.3)	394 (7.8)	367 (9.3)	*7337*
	**CC**	342 (9.1)	361 (9.7)	442 (10.7)	462 (10.8)	563 (13.1)	462 (10.0)	469 (9.7)	505 (10.5)	509 (10.5)	477 (9.3)	463 (9.2)	466 (9.0)	486 (9.1)	525 (10.3)	477 (12.1)	*7009*
	**Other**	229 (6.1)	243 (6.6)	267 (6.4)	229 (5.3)	245 (5.7)	274 (5.9)	313 (6.5)	266 (5.6)	305 (6.3)	308 (6.0)	314 (6.2)	301 (5.9)	334 (6.3)	315 (6.2)	267 (6.8)	*4210*
	Total	*3756*	*3707*	*4131*	*4284*	*4307*	*4639*	*4842*	*4790*	*4833*	*5116*	*5047*	*5152*	*5322*	*5078*	*3946*	***68950***
	**Pure NASH**	7 (0.2)	7 (0.2)	11 (0.3)	18 (0.4)	28 (0.7)	26 (0.6)	40 (0.8)	56 (1.2)	66 (1.4)	106 (2.1)	98 (1.9)	144 (2.8)	186 (3.5)	194 (3.8)	184 (4.7)	**1171**
	**Pres' NASH**	38 (1.0)	46 (1.2)	33 (0.8)	54 (1.3)	78 (1.7)	106 (2.2)	135 (2.8)	122 (2.5)	139 (2.8)	113 (2.2)	134 (2.7)	130 (2.5)	145 (2.7)	149 (3.0)	148 (3.7)	**1570**

Table S1. Trends of annual primary LTs performed for different indications in the ELTR region. Both absolute numbers and percentages (in brackets) are reported. Additional figures for patients classified as "pure NASH" (patients coded as NASH in the ELTR) and "pres' NASH" (presumed NASH = patients coded as cryptogenic cirrhosis with a BMI > 30kg.m^{-2} in the ELTR) are also provided. *Data from 2016 is incomplete, as not all centres had entered data on to ELTR at the time of collection

	Odds ratio of receiving a DCD organ	
	**OR (95% CI)**	***P***
**NASH**	1.53 (0.21-11.11)	0.677
**Era of transplant**		**<0.001**
2002-2004	1.00	
2005-2007	2.12 (1.54-2.92)	**<0.001**
2008-2010	5.10 (3.81-6.83)	**<0.001**
2011-2013	7.60 (5.72-10.10)	**<0.001**
2014-2016	12.18 (9.20-16.12)	**<0.001**
**Era of transplant * NASH (interaction term)**		0.765
2002-2004*NASH	1.00	
2005-2007*NASH	1.99 (0.24-16.25)	0.521
2008-2010*NASH	1.19 (0.57-9.07)	0.867
2011-2013*NASH	1.23 (0.17-9.19)	0.839
2014-2016*NASH	1.32 (0.18-9.71)	0.787

Table S2. Interaction analysis between NASH and era of transplant on the rate of DCD transplants. Binary logistic model: the interaction term (Era of transplant*NASH) tests whether the difference in the DCD rate between NASH and non-NASH varies with time. Bold *P*-values are significant at *P*<0.05

	Recipients without HCC			Recipients with HCC		
	**NASH** N=1667	**Non-NASH** N=47087	***P***	**NASH** N=1073	**Non-NASH** N=19122	***P***
**RECIPIENT CHARACTERISTICS**						
**Age; years; *median (IQR)***	58 (51-63)	53 (46-59)	**<0.001**	61 (57-65)	58 (53-63)	**<0.001**
**Sex: male; *%***	64.4	67.3	**0.011**	81.6	83.8	0.061
**Blood group; *%***			0.875			0.076
A	43.8	43.3		43.2	44.3	
AB	5.8	5.8		5.9	5.1	
B	12.4	13.1		14.0	11.7	
O	38.0	37.8		37.0	38.9	
**BMI; kg.m^{-2}; *mean (SD)***	32.6 (4.91)	25.6 (4.57)	**<0.001**	32.6 (4.98)	26.4 (4.05)	**<0.001**
**MELD; *median (IQR)***	18 (14-23)	18 (13-26)	**0.019**	13 (9-17)	12 (9-16)	**0.016**
**DONOR CHARACTERISTICS**						
**Age; *median (IQR)***	51 (38-62)	50 (36-63)	0.522	55 (41-68)	55 (44-67)	0.553
**Sex: male; *%***	61.1	57.1	**0.001**	64.2	58.7	**<0.001**
**Blood group; *%***			0.170			0.229
A	41.9	42.4		41.7	43.3	
AB	4.4	4.3		4.7	3.9	
B	10.4	12.0		12.5	11.0	
O	43.4	41.3		41.1	41.7	
**BMI; kg.m^{-2}; *mean (SD)***	26.8 (4.70)	25.4 (4.26)	**<0.001**	27.1 (4.96)	25.7 (4.38)	**<0.001**
**Type of donor; *%***			**<0.001**			**<0.001**
DBD	82.4	90.4		88.0	90.3	
DCD	6.1	2.5		7.3	2.8	
Domino	0.5	0.7		1.2	1.6	
Living	10.9	6.4		3.5	5.2	

Table S3. Donor and recipient factors in patients transplanted for NASH and non-NASH indications described by the absence or presence of concomitant HCC. Bold *P*-values are significant at *P*<0.05

	Univariable	Multivariable
	**HR (95% CI)**	**HR (95% CI)**
**RECIPIENT CHARACTERISTICS**		
NASH (vs non-NASH)	*1.09 (0.97-1.23)*	*1.10 (0.97-1.24)*
**Cirrhosis aetiology**	**Overall****	n/a
NASH	1.00	
ARLD	**0.87 (0.76-0.99)***	
HCV	1.07 (0.94-1.21)	
AiLD	**0.76 (0.58-0.98)***	
HBV	**0.68 (0.59-0.79)****	
CC	0.93 (0.81-1.06)	
Other	**0.74 (0.62-0.88)***	
**Age (years)**	**Overall****	**Overall****
</=45	1.00	1.00
46-55	**1.14 (1.01-1.29)***	**1.18 (1.05-1.34)***
56-60	**1.27 (1.13-1.44)****	**1.33 (1.18-1.51)****
61-65	**1.42 (1.26-1.60)***	**1.51 (1.33-1.70)****
>65	**1.52 (1.34-1.73)***	**1.67 (1.46-1.91)****
**Sex: male**	0.97 (0.90-1.04)	not in final model
**MELD**	**Overall****	**Overall****
≤11	1.00	1.00
>11, ≤14	0.93 (0.84-1.02)	0.94 (0.85-1.03)
>14, ≤18	0.94 (0.85-1.03)	0.93 (0.85-1.03)
>18, ≤23	1.03 (0.92-1.16)	1.04 (0.92-1.17)
>23	**1.40 (1.24-1.59)****	**1.41 (1.25-1.60)****
Missing value	**1.17 (1.09-1.25)****	**1.24 (1.14-1.36)****
**Blood group**	**Overall**	not in final model
A	1.00	
AB	0.94 (0.83-1.06)	
B	0.95 (0.97-1.04)	
O	1.02 (0.96-1.08)	
**BMI (kg.m^{-2})**	**Overall**	not in final model
≤18.5	1.11 (0.81-1.51)	
>18.5, ≤25.0	1.00	
>25.0, ≤30.0	0.97 (0.89-1.05)	
>30.0, ≤35.0	1.02 (0.93-1.14)	
>35.0, ≤40.0	0.90 (0.74-1.10)	
>40.0	1.13 (0.77-1.67)	
Missing value	1.01 (0.94-1.09)	
**DONOR CHARACTERISTICS**		
**Age (years)**	**Overall****	**Overall****
</=34	1.00	1.00
35-47	1.03 (0.94-1.13)	1.03 (0.94-1.13)
48-57	1.09 (1.00-1.19)	**1.10 (1.00-1.20)***
58-67	**1.17 (1.07-1.27)****	**1.18 (1.07-1.29)***

>68	**1.19 (1.09-1.29)****	**1.19 (1.09-1.30)****
**Sex: male**	0.95 (0.90-1.00)	not in final model
**Blood group**	**Overall**	not in final model
A	1.00	
AB	0.90 (0.78-1.04)	
B	0.96 (0.88-1.05)	
O	1.02 (0.97-1.08)	
**BMI (kg.m^{-2})**	**Overall**	**Overall****
≤18.5	1.01 (0.82-1.26)	0.99 (0.79-1.24)
>18.5, ≤25.0	1.00	1.00
>25.0, ≤30.0	1.07 (0.99-1.14)	1.06 (0.99-1.14)
>30.0, ≤35.0	**1.13 (1.01-1.26)***	**1.13 (1.01-1.27)***
>35.0, ≤40.0	**1.23 (1.00-1.51)***	**1.28 (1.04-1.58)***
>40.0	1.05 (0.75-1.47)	1.11 (0.79-1.57)
Missing value	1.02 (0.95-1.09)	0.94 (0.87-1.01)
**Type of donor**	**Overall****	**Overall****
DBD	1.00	1.00
DCD	1.01 (0.85-1.20)	1.09 (0.91-1.29)
Domino	1.08 (0.89-1.31)	1.09 (0.90-1.32)
Living	**1.25 (1.11-1.40)****	**1.45 (1.28-1.64)****
**OTHER SIGNIFICANT VARIABLES**		
**Re-transplant**	**1.78 (1.63-1.95)****	**1.81 (1.65-1.98)****
**Era of transplant**	**Overall****	**Overall***
2002-2004	1.00	1.00
2005-2007	1.00 (0.93-1.08)	1.05 (0.96-1.13)
2008-2010	0.95 (0.87-1.03)	1.02 (0.93-1.11)
2011-2013	**0.85 (0.78-0.93)****	0.93 (0.84-1.04)
2014-2016	**0.77 (0.68-0.86)****	**0.84 (0.73-0.95)***

Table S4. Recipient and donor factors that influence patient survival in transplant recipients with HCC. The final multivariable models were based on 19,514 patients. *P<0.05; **P<0.001

	Patient survival				Overall allograft survival			
	Univariable		Multivariable		Univariable		Multivariable	
	HR (95% CI)	*P*	HR (95% CI)	*P*	HR (95% CI)	*P*	HR (95% CI)	*P*
**RECIPIENT CHARACTERISTICS**								
NASH (vs non-NASH)	*1.12 (1.04-1.22)*	***0.005***	*1.02 (0.93-1.11)*	*0.713*	*1.06 (0.98-1.14)*	*0.132*	*0.99 (0.91-1.08)*	*0.815*
**Aetiology of cirrhosis**		**<0.001**	n/a			**<0.001**	n/a	
NASH	1.00				1.00			
ARLD	0.89 (0.82-0.97)	**0.006**			0.92 (0.85-0.99)	**0.034**		
HCV	1.19 (1.09-1.29)	**<0.001**			1.21 (1.12-1.31)	**<0.001**		
AiLD	0.58 (0.53-0.63)	**<0.001**			0.75 (0.69-0.81)	**<0.001**		
HBV	0.68 (0.62-0.75)	**<0.001**			0.73 (0.67-0.80)	**<0.001**		
CC	0.99 (0.91-1.09)	0.890			1.02 (0.93-1.11)	0.732		
Other	0.68 (0.61-0.76)	**<0.001**			0.76 (0.69-0.83)	**<0.001**		
**Age (years)**		**<0.001**		**<0.001**		**<0.001**		**<0.001**
</=45	1.00		1.00		1.00		1.00	
46-55	1.26 (1.20-1.32)	**<0.001**	1.25 (1.19-1.32)	**<0.001**	1.13 (1.08-1.17)	**<0.001**	1.08 (1.04-1.13)	**<0.001**
56-60	1.48 (1.41-1.56)	**<0.001**	1.48 (1.41-1.56)	**<0.001**	1.24 (1.19-1.30)	**<0.001**	1.18 (1.13-1.24)	**<0.001**
61-65	1.73 (1.65-1.82)	**<0.001**	1.72 (1.63-1.82)	**<0.001**	1.39 (1.33-1.46)	**<0.001**	1.31 (1.25-1.37)	**<0.001**
>65	1.90 (1.78-2.01)	**<0.001**	1.94 (1.82-2.07)	**<0.001**	1.49 (1.41-1.58)	**<0.001**	1.42 (1.34-1.51)	**<0.001**
**Sex: male**	1.12 (1.08-1.16)	**<0.001**	1.08 (1.04-1.12)	**<0.001**	1.09 (1.06-1.13)	**<0.001**	1.07 (1.03-1.10)	**<0.001**
**MELD**		**<0.001**		**<0.001**		**<0.001**		**<0.001**
≤11	1.00		1.00		1.00		1.00	
>11, ≤14	0.90 (0.85-0.96)	**0.002**	0.96 (0.90-1.03)	0.230	0.91 (0.86-0.97)	**0.003**	0.95 (0.90-1.01)	0.091
>14, ≤18	0.90 (0.85-0.96)	**<0.001**	1.00 (0.94-1.06)	0.913	0.91 (0.86-0.96)	**<0.001**	0.97 (0.92-1.03)	0.270
>18, ≤23	0.91 (0.86-0.97)	**0.005**	1.05 (1.05-1.12)	0.164	0.90 (0.85-0.95)	**<0.001**	0.98 (0.92-1.04)	0.438
>23	1.30 (1.23-1.38)	**<0.001**	1.52 (1.52-1.62)	**<0.001**	1.24 (1.17-1.30)	**<0.001**	1.36 (1.29-1.44)	**<0.001**
Missing value	1.08 (1.03-1.13)	**0.002**	1.24 (1.24-1.32)	**<0.001**	1.06 (1.01-1.11)	**0.010**	1.25 (1.18-1.32)	**<0.001**
**Blood group**		0.393	not in final model			0.763		**0.012**
A	1.00				1.00		1.00	
AB	0.96 (0.90-1.03)	0.246			0.97 (0.91-1.04)	0.385	1.18 (1.05-1.33)	**0.008**
B	0.98 (0.93-1.02)	0.304			0.98 (0.94-1.03)	0.480	1.07 (0.95-1.22)	0.262
O	1.01 (0.97-1.04)	0.705			1.00 (0.97-1.03)	0.905	0.96 (0.88-1.06)	0.417
**BMI (kg.m^{-2})**		**0.002**		**<0.001**		**0.005**		**<0.001**
≤18.5	1.15 (1.03-1.29)	**0.017**	1.30 (1.16-1.47)	**<0.001**	1.17 (1.05-1.30)	**0.005**	1.29 (1.15-1.44)	**<0.001**
>18.5, ≤25.0	1.00		1.00		1.00		1.00	
>25.0, ≤30.0	1.01 (0.97-1.06)	0.608	0.95 (0.91-0.99)	**0.023**	0.98 (0.94-1.02)	0.359	0.93 (0.89-0.97)	**0.001**
>30.0, ≤35.0	1.09 (1.03-1.15)	**0.003**	1.02 (0.96-1.08)	0.582	1.03 (0.98-1.09)	0.232	0.99 (0.93-1.04)	0.601
>35.0, ≤40.0	1.04 (0.94-1.16)	0.460	0.99 (0.89-1.11)	0.894	0.96 (0.87-1.06)	0.417	0.93 (0.84-1.03)	0.137
>40.0	1.30 (1.08-1.57)	**0.005**	1.26 (1.06-1.55)	**0.010**	1.21 (1.01-1.44)	**0.038**	1.19 (1.00-1.42)	0.056
Missing value	1.04 (1.00-1.08)	0.068	1.00 (0.94-1.05)	0.889	0.99 (0.95-1.02)	0.410	0.94 (0.89-0.99)	**0.021**
**HCC**	1.25 (1.21-1.29)	**<0.001**	1.18 (1.14-1.22)	**<0.001**	1.17 (1.14-1.21)	**<0.001**	1.13 (1.09-1.17)	**<0.001**
**DONOR CHARACTERISTICS**								
**Age (years)**		**<0.001**		**<0.001**		**<0.001**		**<0.001**
</=34	1.00		1.00		1.00		1.00	
35-47	1.15 (1.10-1.21)	**<0.001**	1.12 (1.07-1.18)	**<0.001**	1.16 (1.11-1.22)	**<0.001**	1.14 (1.09-1.20)	**<0.001**
48-57	1.24 (1.19-1.30)	**<0.001**	1.21 (1.15-1.27)	**<0.001**	1.26 (1.21-1.32)	**<0.001**	1.26 (1.20-1.32)	**<0.001**
58-67	1.39 (1.32-1.45)	**<0.001**	1.32 (1.26-1.39)	**<0.001**	1.40 (1.34-1.47)	**<0.001**	1.39 (1.33-1.46)	**<0.001**
>68	1.51 (1.44-1.58)	**<0.001**	1.40 (1.33-1.48)	**<0.001**	1.54 (1.47-1.61)	**<0.001**	1.53 (1.46-1.60)	**<0.001**
**Sex: male**	0.98 (0.95-1.01)	0.221	not in final model		0.97 (0.94-0.99)	**0.031**	not in final model	
**Blood group**		0.314	not in final model			**0.027**		**0.010**
A	1.00				1.00		1.00	
AB	0.91 (0.84-0.98)	**0.014**			0.91 (0.85-0.98)	**0.017**	0.82 (0.72-0.95)	**0.006**
B	0.96 (0.91-1.01)	0.091			0.97 (0.92-1.01)	0.149	0.93 (0.81-1.05)	0.238
O	1.02 (0.99-1.05)	0.272			1.01 (0.98-1.04)	0.590	1.04 (0.95-1.14)	0.421
**BMI (kg.m^{-2})**		**<0.001**		**<0.001**		**<0.001**		**<0.001**

≤18.5	0.94 (0.84-1.06)	0.317	0.92 (0.82-1.06)	0.263	0.97 (0.87-1.08)	0.566	0.97 (0.86-1.09)	0.559
>18.5, ≤25.0	1.00		1.00		1.00		1.00	
>25.0, ≤30.0	1.09 (1.05-1.13)	**<0.001**	1.03 (0.99-1.08)	0.114	1.11 (1.11-1.07)	**<0.001**	1.06 (1.02-1.10)	**0.004**
>30.0, ≤35.0	1.11 (1.04-1.18)	**0.001**	1.05 (0.99-1.13)	0.111	1.11 (1.04-1.18)	**0.001**	1.06 (1.00-1.13)	**0.044**
>35.0, ≤40.0	1.15 (1.02-1.30)	**0.022**	1.10 (0.98-1.25)	0.106	1.12 (1.00-1.26)	**0.049**	1.10 (0.98-1.23)	0.126
>40.0	0.95 (0.77-1.16)	0.616	0.92 (0.76-1.14)	0.475	0.94 (0.78-1.14)	0.530	0.93 (0.77-1.13)	0.467
Missing value	0.96 (0.93-0.99)	**0.037**	0.89 (0.85-0.94)	**<0.001**	0.94 (0.91-0.98)	**0.001**	0.87 (0.83-0.91)	**<0.001**
**Type of donor**		**<0.001**		**<0.001**		**<0.001**		**<0.001**
DBD	1.00		1.00		1.00		1.00	
DCD	0.80 (0.71-0.89)	**<0.001**	0.85 (0.76-0.96)	**0.006**	0.98 (0.89-1.08)	0.719	1.07 (0.97-1.18)	0.191
Domino	1.19 (1.05-1.36)	**0.009**	1.14 (1.00-1.31)	0.052	1.20 (1.06-1.36)	**0.005**	1.24 (1.09-1.40)	**0.001**
Living	1.17 (1.10-1.25)	**<0.001**	1.47 (1.37-1.57)	**<0.001**	1.12 (1.06-1.19)	**<0.001**	1.36 (1.27-1.46)	**<0.001**
**OTHER CHARACTERISTICS**								
**Re-transplant**	1.74 (1.66-1.83)	**<0.001**	1.80 (1.72-1.89)	**<0.001**	n/a			
**Era of transplant**		**<0.001**		**<0.001**		**<0.001**		**<0.001**
2002-2004	1.00		1.00		1.00		1.00	
2005-2007	1.05 (1.01-1.10)	**0.019**	1.04 (0.99-1.09)	0.063	1.04 (1.00-1.09)	**0.042**	1.04 (0.99-1.08)	0.088
2008-2010	1.10 (1.05-1.15)	**<0.001**	1.05 (1.00-1.11)	**0.047**	1.07 (1.03-1.12)	**0.001**	1.04 (0.99-1.09)	0.110
2011-2013	0.98 (0.98-1.03)	0.427	0.93 (0.88-0.99)	**0.021**	0.98 (0.94-1.03)	0.443	0.94 (0.89-0.99)	**0.034**
2014-2016	0.85 (0.85-0.91)	**<0.001**	0.82 (0.76-0.88)	**<0.001**	0.87 (0.84-0.94)	**<0.001**	0.85 (0.80-0.91)	**<0.001**

Table S5. Recipient and donor factors that influence patient and overall allograft survival in all primary transplant recipients. Missing values were treated as a separate category for MELD, recipient BMI and donor BMI. Significant determinants of were analysed by univariable Cox regression, and independent predictors were sought in a multivariable Cox regression model with a backwards stepwise approach. NASH was not selected for inclusion in the final regression models, and so was entered into models alongside the factors identified as significant. The final multivariable models were based on 66,672 patients for patient survival, and 66,491 patients for graft survival. Bold *P* values are significant at *P*<0.05.

	Recipients without HCC				Recipients with HCC			
	Univariable		Multivariable		Univariable		Multivariable	
	HR (95% CI)	*P*	HR (95% CI)	*P*	HR (95% CI)	*P*	HR (95% CI)	*P*
**RECIPIENT CHARACTERISTICS**								
NASH (vs non-NASH)	*1.06 (0.96-1.17)*	*0.082*	*0.98 (0.88-1.09)*	*0.644*	*1.02 (0.91-1.15)*	*0.156*	*1.02 (0.90-1.15)*	*0.797*
**Aetiology of cirrhosis**		**<0.001**	n/a			**<0.001**	n/a	
NASH	1.00				1.00			
ARLD	0.95 (0.86-1.05)	0.291			0.93 (0.82-1.05)	0.240		
HCV	1.27 (1.14-1.40)	**<0.001**			1.14 (1.01-1.28)	**0.040**		
AiLD	0.78 (0.70-0.86)	**<0.001**			0.88 (0.69-1.11)	0.278		
HBV	0.72 (0.64-0.80)	**<0.001**			0.75 (0.65-0.86)	**<0.001**		
CC	1.01 (0.90-1.13)	0.892			0.99 (0.87-1.12)	0.842		
Other	0.76 (0.67-0.85)	**<0.001**			0.82 (0.70-0.98)	**0.025**		
**Age (years)**		**<0.001**		**<0.001**		**<0.001**		**<0.001**
</=45	1.00		1.00		1.00		1.00	
46-55	1.10 (1.05-1.16)	**<0.001**	1.07 (1.02-1.12)	**0.009**	1.08 (0.97-1.21)	0.167	1.09 (0.98-1.22)	0.126
56-60	1.23 (1.17-1.29)	**<0.001**	1.18 (1.12-1.25)	**<0.001**	1.14 (1.02-1.27)	**0.024**	1.14 (1.02-1.28)	**0.023**
61-65	1.40 (1.32-1.48)	**<0.001**	1.33 (1.26-1.41)	**<0.001**	1.22 (1.10-1.37)	**<0.001**	1.22 (1.09-1.37)	**<0.001**
>65	1.53 (1.43-1.64)	**<0.001**	1.48 (1.38-1.59)	**<0.001**	1.28 (1.13-1.44)	**<0.001**	1.30 (1.15-1.47)	**<0.001**
**Sex: male**	1.10 (1.06-1.14)	**<0.001**	1.09 (1.05-1.13)	**<0.001**	0.97 (0.91-1.03)	0.324	not in final model	
**MELD**		**<0.001**		**<0.001**		**<0.001**		**<0.001**
≤11	1.00		1.00		1.00		1.00	
>11, ≤14	0.95 (0.87-1.03)	0.183	0.94 (0.87-1.02)	0.142	0.93 (0.85-1.02)	0.118	0.94 (0.85-1.02)	0.133
>14, ≤18	0.94 (0.87-1.01)	0.078	0.95 (0.88-1.02)	0.164	0.97 (0.89-1.05)	0.420	0.96 (0.88-1.05)	0.341
>18, ≤23	0.93 (0.86-1.00)	0.061	0.94 (0.87-1.02)	0.144	1.00 (0.89-1.11)	0.964	0.99 (0.89-1.11)	0.862
>23	1.31 (1.31-1.40)	**<0.001**	1.31 (1.22-1.40)	**<0.001**	1.37 (1.22-1.54)	**<0.001**	1.36 (1.21-1.54)	**<0.001**
Missing value	1.07 (1.07-1.15)	**0.032**	1.20 (1.11-1.30)	**<0.001**	1.15 (1.08-1.23)	**<0.001**	1.26 (1.15-1.37)	**<0.001**
**Blood group**		0.967		**0.002**		0.565	not in final model	
A	1.00		1.00		1.00			
AB	0.99 (0.92-1.07)	0.803	1.21 (1.05-1.40)	**0.008**	0.94 (0.83-1.06)	0.314		
B	0.99 (0.94-1.05)	0.817	1.13 (0.97-1.30)	0.109	0.97 (0.89-1.05)	0.477		
O	0.99 (0.96-1.03)	0.628	0.94 (0.84-1.05)	0.252	1.01 (0.96-1.07)	0.690		
**BMI (kg.m^{-2})**		**0.001**		**<0.001**		0.359	not in final model	
≤18.5	1.69 (1.04-1.31)	**0.007**	1.26 (1.12-1.42)	**<0.001**	1.28 (0.97-1.70)	0.084		
>18.5, ≤25.0	1.00		1.00		1.00			
>25.0, ≤30.0	0.97 (0.92-1.01)	0.166	0.92 (0.88-0.97)	**0.001**	0.98 (0.91-1.05)	0.540		
>30.0, ≤35.0	1.03 (0.96-1.09)	0.444	0.98 (0.92-1.05)	0.642	1.02 (0.93-1.12)	0.683		
>35.0, ≤40.0	0.98 (0.87-1.10)	0.770	0.96 (0.85-1.08)	0.450	0.89 (0.73-1.07)	0.210		
>40.0	1.25 (1.03-1.53)	**0.025**	1.25 (1.02-1.53)	**0.029**	1.03 (0.70-1.51)	0.884		
Missing value	0.96 (0.92-0.99)	**0.031**	0.94 (0.88-1.00)	**0.047**	1.02 (0.95-1.09)	0.623		
**DONOR CHARACTERISTICS**								
**Age (years)**		**<0.001**		**<0.001**		**<0.001**		**<0.001**
</=34	1.00		1.00		1.00		1.00	
35-47	1.20 (1.13-1.27)	**<0.001**	1.18 (1.11-1.24)	**<0.001**	1.05 (0.96-1.14)	0.275	1.05 (0.97-1.15)	0.237
48-57	1.31 (1.24-1.38)	**<0.001**	1.30 (1.23-1.37)	**<0.001**	1.13 (1.04-1.23)	**0.004**	1.16 (1.06-1.26)	**0.001**
58-67	1.46 (1.46-1.54)	**<0.001**	1.45 (1.37-1.53)	**<0.001**	1.22 (1.12-1.33)	**<0.001**	1.25 (1.15-1.37)	**<0.001**
>68	1.65 (1.65-1.74)	**<0.001**	1.65 (1.56-1.75)	**<0.001**	1.26 (1.16-1.36)	**<0.001**	1.31 (1.20-1.42)	**<0.001**
**Sex: male**	0.97 (0.94-1.00)	0.051	not in final model		0.97 (0.92-1.02)	0.178	not in final model	
**Blood group**		0.145		**0.003**		0.334	not in final model	
A	1.00		1.00		1.00			
AB	0.93 (0.85-1.01)	0.087	0.81 (0.69-0.96)	**0.013**	0.89 (0.78-1.02)	0.097		
B	0.96 (0.91-1.02)	0.175	0.88 (0.76-1.03)	0.109	0.99 (0.91-1.07)	0.749		
O	1.01 (0.97-1.04)	0.748	1.06 (0.95-1.19)	0.278	1.01 (0.96-1.07)	0.698		
**BMI (kg.m^{-2})**		**<0.001**		**<0.001**		0.281		**0.001**
≤18.5	0.94 (0.83-1.07)	0.364	0.94 (0.82-1.08)	0.400	1.05 (0.85-1.28)	0.667	1.03 (0.84-1.27)	0.781
>18.5, ≤25.0	1.00		1.00		1.00		1.00	
>25.0, ≤30.0	1.12 (1.08-1.17)	**<0.001**	1.05 (1.01-1.10)	**0.018**	1.07 (0.99-1.14)	0.065	1.05 (0.98-1.13)	0.160

>30.0, ≤35.0	1.11 (1.03-1.19)	**0.006**	1.05 (0.98-1.13)	0.161	1.09 (0.98-1.21)	0.114	1.07 (0.97-1.19)	0.192
>35.0, ≤40.0	1.10 (0.96-1.27)	0.176	1.06 (0.92-1.22)	0.432	1.14 (0.94-1.39)	0.189	1.16 (0.95-1.42)	0.151
>40.0	0.88 (0.69-1.11)	0.277	0.85 (0.67-1.08)	0.192	1.07 (0.78-1.47)	0.687	1.12 (0.81-1.53)	0.500
Missing value	0.91 (0.87-0.95)	<0.001	0.84 (0.80-0.89)	**<0.001**	1.00 (0.94-1.07)	0.961	0.89 (0.83-0.96)	**0.002**
**Type of donor**		**0.001**		**<0.001**		0.078		**<0.001**
DBD	1.00		1.00		1.00		1.00	
DCD	0.91 (0.81-1.02)	0.123	0.98 (0.87-1.11)	0.783	1.12 (0.96-1.30)	0.147	1.23 (1.05-1.43)	**0.010**
Domino	1.21 (1.02-1.44)	**0.032**	1.30 (1.08-1.55)	**0.004**	1.10 (0.92-1.32)	0.287	1.15 (0.96-1.38)	0.127
Living	1.12 (1.04-1.20)	**0.003**	1.37 (1.26-1.48)	**<0.001**	1.13 (1.00-1.26)	**0.046**	1.30 (1.15-1.47)	**<0.001**
**OTHER VARIABLES**								
**Era of transplant**		**<0.001**		**<0.001**		**<0.001**		**0.012**
2002-2004	1.00		1.00		1.00		1.00	
2005-2007	1.04 (0.99-1.09)	0.125	1.03 (0.98-1.08)	0.251	1.00 (0.93-1.08)	0.908	1.04 (0.96-1.12)	0.361
2008-2010	1.10 (1.04-1.15)	**<0.001**	1.04 (0.98-1.10)	0.166	0.96 (0.89-1.04)	0.293	1.02 (0.94-1.11)	0.646
2011-2013	1.00 (0.95-1.06)	0.970	0.93 (0.87-0.99)	**0.029**	0.88 (0.81-0.95)	**0.002**	0.96 (0.87-1.06)	0.416
2014-2016	0.90 (0.85-0.97)	**<0.001**	0.85 (0.78-0.91)	**<0.001**	0.81 (0.73-0.89)	**<0.001**	0.87 (0.77-0.98)	**0.019**

Table S6. Recipient and donor factors that influence overall allograft survival in transplant recipients. Missing values were treated as a separate category for MELD, recipient BMI and donor BMI. Determinants of graft survival were analysed by univariable Cox regression. Independent predictors were sought in a multivariable Cox regression model with a backwards stepwise approach. NASH was not selected for inclusion in the final regression models, and so was entered into models alongside the factors identified as significant. The final multivariable models were based on 47,040 patients for those without HCC, and 19,514 patients for those with HCC. Bold *P* values are significant at *P*<0.05

Causes of deaths in transplant recipients (without HCC)										
N; (%)	**ARLD** N=4664	**HCV** N=3022	**AiLD** N=1523	**HBV** N=770	**CC** N=980	**Other** N=645	**Non-NASH cumulative** N=11604	**NASH** N=359	**HR (CI)**	***P***
**Infection**	1011 (21.7)	629 (20.8)	347 (22.8)	179 (23.2)	197 (20.1)	149 (23.1)	2512 (21.6)	86 (24.0)	1.15 (0.92-1.42)	0.216
**Cardio/cerebrovascular complication**	417 (8.9)	245 (8.1)	104 (6.8)	54 (7.0)	61 (6.2)	56 (8.7)	937 (8.1)	19 (5.3)	0.70 (0.44-1.10)	0.123
**Primary non-function or dysfunction**	128 (2.7)	96 (3.2)	63 (4.1)	37 (4.8)	33 (3.4)	21 (3.3)	378 (3.3)	16 (4.5)	1.26 (0.77-2.08)	0.360
**Rejection (acute or chronic)**	111 (2.4)	73 (2.4)	49 (3.2)	22 (2.9)	30 (3.1)	17 (2.6)	302 (2.6)	14 (3.9)	1.69 (0.99-2.89)	0.055
**Gastrointestinal complications**	132 (2.8)	102 (3.4)	35 (2.3)	19 (2.5)	30 (3.1)	15 (2.3)	333 (2.9)	10 (2.8)	0.73 (0.32-1.64)	0.967
**Malignancy – solid organ extrahepatic**	603 (12.9)	122 (4.0)	101 (6.6)	52 (6.8)	42 (4.3)	37 (5.7)	957 (8.2)	9 (2.5)	0.41 (0.21-0.79)	**0.008**
**Other liver complication**	116 (2.5)	68 (2.3)	47 (3.1)	20 (2.6)	27 (2.8)	20 (3.1)	298 (2.6)	7 (1.9)	0.79 (0.37-1.67)	0.534
**Biliary complications**	93 (2.0)	55 (1.8)	46 (3.0)	28 (3.6)	14 (1.4)	10 (1.6)	246 (2.1)	6 (1.7)	0.84 (0.37-1.88)	0.663
**Vascular complications**	115 (2.5)	46 (1.5)	31 (2.0)	31 (4.0)	24 (2.4)	13 (2.0)	260 (2.2)	6 (1.7)	0.73 (0.32-1.64)	0.444
**Malignancy – recurrence of HCC**	55 (1.2)	66 (2.2)	42 (2.8)	19 (2.5)	16 (1.6)	7 (1.1)	205 (1.8)	6 (1.7)	1.11 (0.49-2.51)	0.798
**Malignancy – lymphoproliferative**	40 (0.9)	34 (1.1)	25 (1.6)	6 (0.8)	5 (0.5)	13 (2.0)	123 (1.1)	5 (1.4)	1.65 (0.67-4.03)	0.274
**Intraoperative**	49 (1.1)	39 (1.3)	16 (1.1)	13 (1.7)	10 (1.0)	9 (1.4)	136 (1.2)	5 (1.4)	1.06 (0.43-2.58)	0.902
**Recurrence of 1° liver pathology (non-malignant)**	190 (4.1)	651 (21.5)	42 (2.8)	28 (3.6)	12 (1.2)	12 (1.9)	935 (8.1)	2 (0.6)	0.08 (0.02-0.33)	**<0.001**
Other	146 (3.1)	112 (3.7)	45 (3.0)	35 (4.5)	44 (4.5)	24 (3.7)	406 (3.5)	9 (2.5)	0.76 (0.39-1.47)	0.418
Unknown	1458 (31.3)	684 (22.6)	530 (34.8)	227 (29.5)	435 (44.4)	242 (37.5)	3576 (30.8)	159 (44.3)	1.61 (1.38-1.89)	**<0.001**

Table S7. Causes of death in patients without HCC undergoing primary LT.

Univariable Cox regression models were used to compare cause-specific mortality rates between NASH and non-NASH recipients. Causes of death were grouped into 15 categories: 1) Intraoperative deaths; 2) Primary non- or dysfunction; 3) Vascular complications (hepatic artery/vein thrombosis, early portal vein thrombosis, outflow impairment); 4) Biliary complications; 5) Recurrence of primary non-malignant liver pathology; 6) Rejection; 7) Other liver complications (massive hemorrhagic necrosis, de-novo viral hepatitis, other); 8) Malignancy: HCC; 9) Malignancy: extra-hepatic solid organ; 10) Malignancy: lymphoproliferative; 11) Gastrointestinal complications (visceral

perforation, pancreatitis, other); 12) Infection; 13) Cardio/cerebrovascular complication (myocardial infarction, ischaemic stroke, intracranial hemorrhage, other); 14) Other (including any complication with an overall burden of <1% of deaths – kidney failure, venous thromboembolism, trauma, suicide, bone marrow depression, neurological complication, other); 15) Unknown. Causes of death listed in order of frequency in patients transplanted for NASH. Bold *P* values are significant at *P*<0.05.

Causes of deaths in transplant recipients (with HCC)										
N; (%)	**ARLD** N=1228	**HCV** N=2250	**AiLD** N=73	**HBV** N=609	**CC** N=1015	**Other** N=210	**Non-NASH cumulative** N=5385	**NASH** N=272	**HR (CI)**	***P***
**Malignancy – recurrence of HCC**	178 (14.5)	407 (18.1)	7 (9.6)	166 (27.3)	146 (14.4)	33 (15.7)	937 (17.4)	53 (19.5)	1.24 (0.94-1.64)	0.123
**Infection**	187 (15.2)	376 (16.7)	17 (23.3)	74 (12.2)	128 (12.6)	47 (22.4)	829 (15.4)	28 (10.3)	0.69 (0.47-1.00)	**0.049**
**Malignancy – solid organ extrahepatic**	216 (17.6)	146 (6.5)	6 (8.2)	48 (7.9)	126 (12.4)	22 (10.5)	564 (10.5)	18 (6.6)	0.77 (0.48-1.23)	0.277
**Cardio/cerebrovascular complication**	83 (6.8)	124 (5.5)	4 (5.5)	40 (6.6)	44 (4.3)	14 (6.7)	309 (5.7)	13 (4.8)	0.89 (0.51-1.56)	0.693
**Other liver complication**	16 (1.3)	29 (1.3)	1 (1.4)	12 (2.0)	17 (1.7)	5 (2.4)	80 (1.5)	11 (4.0)	2.76 (1.47-5.19)	**0.002**
**Primary non-function or dysfunction**	31 (2.5)	55 (2.4)	4 (5.5)	22 (3.6)	28 (2.8)	2 (1.0)	142 (2.6)	8 (2.9)	1.02 (0.50-2.08)	0.960
**Gastrointestinal complications**	34 (2.8)	49 (2.2)	2 (2.7)	19 (3.1)	24 (2.4)	5 (2.4)	133 (2.5)	7 (2.6)	1.11 (0.52-2.38)	0.788
**Recurrence of 1° liver pathology (non-malignant)**	85 (6.9)	468 (20.8)	3 (4.1)	32 (5.3)	63 (6.2)	6 (2.9)	657 (12.2)	6 (2.2)	0.21 (0.09-0.46)	**<0.001**
**Vascular complications**	22 (1.8)	44 (2.0)	1 (1.4)	13 (2.1)	15 (1.5)	4 (1.9)	99 (1.8)	4 (1.5)	0.76 (0.28-2.07)	0.596
**Rejection (acute or chronic)**	19 (1.5)	36 (1.6)	1 (1.4)	7 (1.1)	11 (1.1)	7 (3.3)	81 (1.5)	4 (1.5)	1.05 (0.38-2.86)	0.928
**Malignancy – lymphoproliferative**	11 (0.9)	25 (1.1)	3 (4.1)	10 (1.6)	3 (0.3)	4 (1.9)	56 (1.0)	3 (1.1)	1.28 (0.40-4.09)	0.681
**Biliary complications**	22 (1.8)	34 (1.5)	1 (1.4)	6 (1.0)	9 (0.9)	2 (1.0)	74 (1.4)	1 (0.4)	0.28 (0.04-2.01)	0.205
**Intraoperative**	9 (0.7)	24 (1.1)	0 (0.0)	4 (0.7)	10 (1.0)	0 (0.0)	47 (0.9)	2 (0.7)	0.79 (0.19-3.26)	0.748
Other	38 (3.1)	63 (2.8)	1 (1.4)	11 (1.8)	37 (3.6)	4 (1.9)	154 (2.9)	7 (2.6)	1.09 (0.48-2.47)	0.846
Unknown	277 (22.6)	370 (16.4)	22 (30.1)	145 (23.8)	354 (34.9)	55 (26.2)	1223 (22.7)	107 (39.3)	1.89 (1.55-2.30)	**<0.001**

Table S8. Causes of death in patients with HCC undergoing primary LT. Univariable Cox regression models were used to compare cause-specific mortality rates between NASH and non-NASH recipients. Causes of death were grouped into 15 categories: 1) Intraoperative deaths; 2) Primary non- or dysfunction; 3) Vascular complications (hepatic artery/vein thrombosis, early portal vein thrombosis, outflow impairment); 4) Biliary complications; 5) Recurrence of primary non-malignant liver pathology; 6) Rejection; 7) Other liver complications (massive hemorrhagic necrosis, de-novo viral hepatitis, other); 8) Malignancy: HCC; 9) Malignancy: extra-hepatic solid organ; 10) Malignancy:

lymphoproliferative; 11) Gastrointestinal complications (visceral perforation, pancreatitis, other); 12) Infection; 13) Cardio/cerebrovascular complication (myocardial infarction, ischaemic stroke, intracranial hemorrhage, other); 14) Other (including any complication with an overall burden of <1% of deaths – kidney failure, venous thromboembolism, trauma, suicide, bone marrow depression, neurological complication, other); 15) Unknown. Causes of death listed in order of frequency in patients transplanted for NASH. Bold *P* values are significant at *P*<0.05.

	Univariable	Multivariable
	**HR (95% CI)**	
**RECIPIENT CHARACTERISTICS**		
**Age (years)**	Overall	not in final model
≤45	1.00	
46-55	1.94 (0.82-4.56)	
56-60	2.30 (1.00-5.29)	
61-65	2.20 (0.96-5.05)	
>65	2.85 (1.23-6.59)	
**Sex: male**	1.03 (0.76-1.40)	not in final model
**MELD**	Overall	not in final model
≤11	1.00	
>11, ≤14	0.59 (0.40-0.86)	
>14, ≤18	0.85 (0.61-1.18)	
>18, ≤23	0.88 (0.57-1.36)	
>23	1.23 (0.80-1.89)	
Missing value	0.75 (0.47-1.19)	
**Blood group**	Overall	not in final model
A	1.00	
AB	1.22 (0.75-1.98)	
B	0.85 (0.58-1.24)	
O	0.93 (0.72-1.22)	
**BMI ($kg.m^{-2}$)**	Overall	not in final model
≤18.5	n/a	
>18.5, ≤25.0	1.94 (0.63-5.95)	
>25.0, ≤30.0	1.00	
>30.0, ≤35.0	1.98 (1.13-3.47)	
>35.0, ≤40.0	1.63 (0.87-3.06)	
>40.0	2.58 (1.15-5.75)	
Missing value	1.94 (0.63-5.95)	
**DONOR CHARACTERISTICS**		
**Age (years)**	Overall	not in final model
≤45	1.00	
46-55	1.26 (0.82-1.93)	
56-60	1.06 (0.71-1.60)	
61-65	1.15 (0.75-1.75)	
>65	1.33 (0.89-1.99)	
**Sex: male**	0.97 (0.76-1.24)	not in final model
**Blood group**	Overall	not in final model
A	1.00	
AB	1.28 (0.76-2.15)	
B	0.74 (0.49-1.12)	
O	0.96 (0.74-1.25)	
**BMI ($kg.m^{-2}$)**	Overall	not in final model
≤18.5	1.23 (0.30-4.96)	

>18.5, ≤25.0	0.99 (0.75-1.30)	
>25.0, ≤30.0	1.00	
>30.0, ≤35.0	0.74 (0.49-1.12)	
>35.0, ≤40.0	1.14 (0.64-2.03)	
>40.0	1.85 (0.90-3.78)	
Missing value	0.66 (0.39-1.09)	
**Type of donor**	Overall	not in final model
DBD	1.00	
DCD	0.78 (0.45-1.34)	
Domino	0.77 (0.25-2.41)	
Living	1.54 (0.84-1.82)	
**OTHER VARIABLES**		
**Era of transplant**	Overall	n/a
2002-2004	1.00	
2005-2007	0.76 (0.43-1.32)	
2008-2010	1.05 (0.62-1.78)	
2011-2013	0.72 (0.41-1.26)	
2014-2016	0.56 (0.31-1.02)	

Table S9. Recipient and donor factors that significantly affect post-transplant survival in patients transplanted for NASH without HCC. The final multivariable models were based on 1,043 patients. *P<0.05; **P<0.001

	**Pure NASH** N=1171	**Presumed NASH** N=1570	***P***
**RECIPIENT CHARACTERISTICS**			
**Age; years; *median (IQR)***	60 (54-65)	59 (53-64)	**<0.001**
**Sex: male; *%***	68.7	72.9	**0.017**
**Blood group; *%***			**0.002**
A	43.6	43.5	
AB	4.0	7.2	
B	12.7	13.3	
O	39.7	36.0	
**BMI; kg.m^{-2}; *mean (SD)***	31.2 (5.2)	33.5 (3.8)	**<0.001**
**MELD; *median (IQR)***	16 (13-21)	15 (11-21)	**0.002**
**HCC; *%***	28.5	47.1	**<0.001**
**DONOR CHARACTERISTICS**			
**Age; years; *median (IQR)***	53 (41-64)	52 (39-63)	0.139
**Sex: male; *%***	59.9	64.1	**0.030**
**Blood group; *%***			**0.003**
A	41.6	42.0	
AB	2.9	5.7	
B	10.9	11.5	
O	44.6	40.9	
**BMI; kg.m^{-2}; *mean (SD)***	26.8 (4.7)	27.0 (4.9)	0.719
**Type of donor; *%***			**<0.001**
DBD	80.5	87.6	
DCD	12.1	2.5	
Domino	1.1	0.6	
Living	6.3	9.2	

Table S10. Donor and recipient factors in patients transplanted for pure NASH and presumed NASH. Comparisons between groups were performed using Mann-Whitney U tests, chi-squared tests and t-tests, as applicable, and bold *P*-values are significant at *P*<0.05.

	**HCC**<br>N (%)
**NASH**	1073 (39.1)
**ARLD**	4717 (21.2)
**HCV**	7118 (44.1)
**AiLD**	320 (3.4)
**HBV**	2857 (38.9)
**CC**	3229 (46.1)
**Other**	881 (20.9)
**Total**	20195 (29.3)

Table S11. The prevalence of HCC in patients transplanted with liver disease of different aetiologies.

	Complete cases<br><br>N=38,084	Cases with missing values*<br>N=30,886	***P***
**RECIPIENT CHARACTERISTICS**			
**Age; years; *median (IQR)***	55 (48-61)	55 (48-61)	**<0.001**
**Sex: male; *%***	71.1	73.3	**<0.001**
**Aetiology**			**<0.001**
NASH	6.1	1.3	
ARLD	30.9	33.9	
HCV	17.7	30.4	
AiLD	15.2	11.4	
HBV	11.1	10.0	
CC	11.0	9.1	
Other	8.0	3.8	
**Blood group; *%***			**<0.001**
A	43.2	44.1	
AB	6.0	5.2	
B	13.3	11.9	
O	37.5	38.8	
**HCC; *%***	28.6	30.1	**<0.001**
**DONOR CHARACTERISTICS**			
**Age; *median (IQR)***	52 (38-64)	52 (38-65)	0.241
**Sex: male; *%***	56.7	59.1	**<0.001**
**Blood group; *%***			**0.001**
A	42.1	43.3	
AB	4.3	4.2	
B	12.0	11.2	
O	41.6	41.2	
**Type of donor; *%* ****			**<0.001**
DBD	86.2	95.1	
DCD	4.7	0.3	
Domino	0.5	1.5	
Living	8.6	3.1	

Table S12. Comparison of donor and recipient factors in patients transplanted for NASH and non-NASH indications. **Missing data points in one of MELD, recipient BMI or donor BMI.*

	Missing indicator analysis<br>N = 47,040	Available case analysis<br>N = 28,643
	**HR (95% CI)**	
**RECIPIENT CHARACTERISTICS**		
NASH (vs non-NASH)	*0.97 (0.86-1.09)*	*1.02 (0.90-1.16)*
**Age (years)**	**Overall****	**Overall****
</=45	1.00	1.00
46-55	**1.24 (1.18-1.31)****	**1.37 (1.27-1.48)****
56-60	**1.49 (1.41-1.59)****	**1.63 (1.50-1.77)****
61-65	**1.78 (1.67-1.89)****	**1.99 (1.82-2.17)****
>65	**2.04 (1.89-2.20)****	**2.19 (1.97-2.42)****
**Sex: male**	**1.11 (1.06-1.15)****	**1.12 (1.06-1.18)****
**MELD**	**Overall****	**Overall****
≤11	1.00	1.00
>11, ≤14	0.98 (0.89-1.07)	0.97 (0.88-1.06)
>14, ≤18	1.02 (0.94-1.11)	1.01 (0.92-1.10)
>18, ≤23	1.05 (0.96-1.15)	1.05 (0.95-1.15)
>23	**1.52 (1.40-1.64)****	**1.54 (1.41-1.67)****
Missing value	**1.22 (1.12-1.33)****	n/a
**Blood group**	**Overall***	**Overall***
A	1.00	1.00
AB	**1.18 (1.01-1.38)***	**1.27 (1.06-1.54)***
B	1.12 (0.95-1.31)	**1.23 (1.01-1.50)***
O	0.94 (0.84-1.07)	1.05 (0.90-1.23)
**BMI ($kg.m^{-2}$)**	**Overall****	**Overall****
≤18.5	**1.34 (1.18-1.52)****	**1.30 (1.13-1.51)****
>18.5, ≤25.0	1.00	1.00
>25.0, ≤30.0	0.95 (0.90-1.01)	0.95 (0.89-1.01)
>30.0, ≤35.0	1.04 (0.97-1.12)	1.02 (0.95-1.11)
>35.0, ≤40.0	1.06 (0.93-1.21)	1.01 (0.88-1.16)
>40.0	**1.35 (1.09-1.67)***	**1.38 (1.10-1.73)***
Missing value	1.04 (0.98-1.11)	n/a
**DONOR CHARACTERISTICS**		
**Age (years)**	**Overall****	**Overall****
</=34	1.00	1.00
35-47	**1.16 (1.09-1.23)****	**1.16 (1.07-1.26)****
48-57	**1.25 (1.18-1.33)****	**1.25 (1.15-1.36)****
58-67	**1.38 (1.29-1.47)****	**1.38 (1.26-1.50)****
>68	**1.52 (1.43-1.62)****	**1.54 (1.41-1.68)****
**Sex: male**	not in final model	not in final model
**Blood group**	**Overall***	**Overall***
A	1.00	1.00
AB	**0.82 (0.68-0.97)***	**0.75 (0.60-0.93)***
B	0.90 (0.76-1.06)	**0.78 (0.64-0.96)***
O	1.07 (0.95-1.21)	0.96 (0.82-1.12)

BMI (kg.m^{-2})	Overall**	
≤18.5	0.91 (0.79-1.06)	not in final model
>18.5, ≤25.0	1.00	
>25.0, ≤30.0	1.02 (0.97-1.07)	
>30.0, ≤35.0	1.02 (0.94-1.11)	
>35.0, ≤40.0	1.02 (0.88-1.19)	
>40.0	0.85 (0.66-1.10)	
Missing value	**0.85 (0.80-0.90)****	n/a
**Type of donor**	**Overall****	**Overall****
DBD	1.00	1.00
DCD	**0.73 (0.62-0.85)****	**0.73 (0.62-0.85)****
Domino	1.20 (0.99-1.45)	1.22 (0.88-1.68)
Living	**1.43 (1.31-1.56)****	**1.59 (1.44-1.76)****
**OTHER VARIABLES**		
**Re-transplant**	**1.80 (1.71-1.91)****	**1.95 (1.81-2.11)****
**Era of transplant**	**Overall****	**Overall****
2002-2004	1.00	1.00
2005-2007	1.03 (0.98-1.09)	**1.18 (1.06-1.30)****
2008-2010	**1.07 (1.00-1.13)***	**1.20 (1.09-1.33)****
2011-2013	**0.93 (0.87-0.99)***	1.03 (0.93-1.15)
2014-2016	**0.81 (0.75-0.89)****	**0.90 (0.80-1.01)***

Table S13. Multivariable analyses by different methods to determine recipient and donor factors that influence patient survival in transplant recipients without HCC.

*P<0.05; **P<0.001

	Missing indicator analysis<br>N = 1,628	Available case analysis<br>N = 1,493
**RECIPIENT CHARACTERISTICS**		
**Age (years)**	**Overall****	**Overall****
≤45	1.00	1.00
46-55	1.31 (0.87-1.98)	1.33 (0.87-2.04)
56-60	1.23 (0.81-1.87)	1.37 (0.89-2.12)
61-65	**2.07 (1.39-3.08)****	**2.20 (1.46-3.31)****
>65	**1.72 (1.10-2.71)***	**1.70 (1.07-2.71)***
**Sex: male**	**0.79 (0.63-0.98)***	**0.78 (0.62-0.98)***
**MELD**	**Overall****	**Overall****
≤11	1.00	1.00
>11, ≤14	1.03 (0.66-1.62)	1.03 (0.67-1.59)
>14, ≤18	0.66 (0.44-1.06)	0.66 (0.44-1.01)
>18, ≤23	0.71 (0.47-1.15)	0.71 (0.46-1.10)
>23	**1.48 (1.04-2.30)***	**1.49 (1.01-2.19)***
Missing value	0.93 (0.57-1.51)	n/a
**Blood group**	not in final model	not in final model
A		
AB		
B		
O		
**BMI (kg.m^{-2})**	**Overall***	**Overall***
≤18.5	**4.29 (1.01-18.21)***	**4.71 (1.11-20.11)***
>18.5, ≤25.0	**2.24 (1.27-3.96)***	**2.13 (1.99-3.80)***
>25.0, ≤30.0	1.00	1.00
>30.0, ≤35.0	1.38 (0.95-2.01)	1.34 (0.92-1.94)
>35.0, ≤40.0	1.43 (0.93-2.18)	1.30 (0.85-1.99)
>40.0	**1.96 (1.16-3.32)***	**1.82 (1.07-3.11)***
Missing value	1.13 (0.49-2.63)	n/a
**DONOR CHARACTERISTICS**		
**Age (years)**	not in final model	not in final model
≤45		
46-55		
56-60		
61-65		
>65		
**Sex: male**	not in final model	not in final model
**Blood group**	**Overall***	**Overall***
A	1.00	1.00
AB	0.99 (0.58-1.70)	1.20 (0.67-1.59)
B	**0.37 (0.22-0.63)****	**0.45 (0.27-0.75)****
O	1.06 (0.85-1.32)	1.06 (0.84-1.34)
**BMI (kg.m^{-2})**	not in final model	not in final model
≤18.5		

>18.5, ≤25.0		
>25.0, ≤30.0		
>30.0, ≤35.0		
>35.0, ≤40.0		
>40.0		
Missing value		
**OTHER VARIABLES**		
**Type of donor**	not in final model	not in final model
DBD		
DCD		
Domino		
Living		

Table S14. Multivariable analyses by different methods to determine recipient and donor factors that significantly affect post-transplant survival in patients transplanted for NASH without HCC. *$P<0.05$; **$P<0.001$
